# Single-cell genomic profiling of human dopamine neurons identifies a population that selectively degenerates in Parkinson’s disease

**DOI:** 10.1038/s41593-022-01061-1

**Published:** 2022-05-05

**Authors:** Tushar Kamath, Abdulraouf Abdulraouf, S. J. Burris, Jonah Langlieb, Vahid Gazestani, Naeem M. Nadaf, Karol Balderrama, Charles Vanderburg, Evan Z. Macosko

**Affiliations:** 1 Broad Institute of Harvard and MIT, Stanley Center for Psychiatric Research, Cambridge, MA USA; 2 Harvard Graduate Program in Biophysics, Harvard University, Cambridge, MA USA; 3 Massachusetts General Hospital, Department of Psychiatry, Boston, MA USA

**Keywords:** Parkinson's disease, Gene expression profiling

## Abstract

The loss of dopamine (DA) neurons within the substantia nigra pars compacta (SNpc) is a defining pathological hallmark of Parkinson’s disease (PD). Nevertheless, the molecular features associated with DA neuron vulnerability have not yet been fully identified. Here, we developed a protocol to enrich and transcriptionally profile DA neurons from patients with PD and matched controls, sampling a total of 387,483 nuclei, including 22,048 DA neuron profiles. We identified ten populations and spatially localized each within the SNpc using Slide-seq. A single subtype, marked by the expression of the gene *AGTR1* and spatially confined to the ventral tier of SNpc, was highly susceptible to loss in PD and showed the strongest upregulation of targets of *TP53* and *NR2F2*, nominating molecular processes associated with degeneration. This same vulnerable population was specifically enriched for the heritable risk associated with PD, highlighting the importance of cell-intrinsic processes in determining the differential vulnerability of DA neurons to PD-associated degeneration.

## Abstract (web summary)

The authors used single-cell genomics to profile thousands of human dopamine neurons and identify one uniquely Parkinson’s disease-susceptible population, which was enriched for genetic risk for Parkinson’s disease.

## Main

The degeneration of midbrain DA neurons within the SNpc is a pathological hallmark of PD and Lewy body dementia (LBD)^1^. Nevertheless, some SNpc neurons are observed to survive even into the late stages of these diseases^2–7^, suggesting differential vulnerability to degeneration. A clearer understanding of the specific molecular characteristics of vulnerable neurons, and the associated cascade of molecular events that lead to their demise, could provide opportunities to refine laboratory models of PD, and aid in the development of disease-modifying or cell-type-specific therapies^8^.

Recent advances in single-cell RNA-sequencing technology^9–11^, and its application to the postmortem human brain, have begun to reveal cell-type-specific changes in several brain diseases^12–16^. While some studies have profiled the human SNpc at single-cell resolution^17–20^, the proportion and absolute number of DA neurons sampled has been small—considerably lower than in murine SNpc—making it challenging to identify molecularly defined subpopulations and robustly compare across subjects.

Here, we developed a protocol for selective enrichment of DA neurons from postmortem human SNpc and used it to define ten transcriptionally-distinct populations by single-nucleus RNA-sequencing (snRNA-seq). We then spatially localized these populations along the dorsal–ventral axis of the SNpc using Slide-seq, a high-resolution spatial transcriptomics technology. Interestingly, we found that one population—marked by expression of *AGTR1*—was highly ventrally localized, consistent with previously identified patterns of DA neuron loss in PD^21^. Additional snRNA-seq analysis of postmortem tissue from patients with PD confirmed the selective loss of this population in PD. Critically, the transcriptional signature of these PD-vulnerable cells was highly enriched for expression of loci associated with PD by previous Genome-Wide Association (GWA) studies. These enrichment results suggest that cell-intrinsic molecular mechanisms play important roles in the selective vulnerability of some DA neuron populations to PD degeneration.

## Results

### A molecular taxonomy of human SNpc DA neurons

**Fig. 1:**
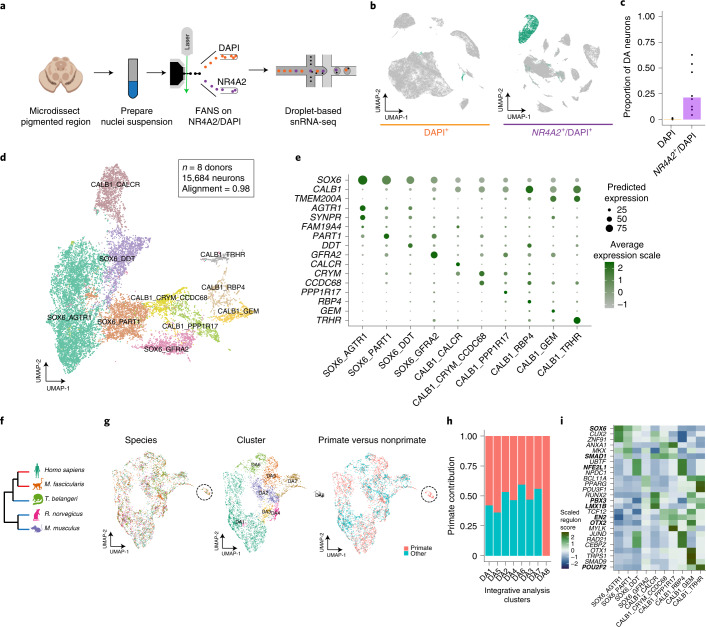
A molecular census of DA neurons in the human substantia nigra. **a**, NR4A2 antibody-based enrichment strategy and snRNA-seq profiling workflow. **b**, UMAP representation of 104,097 *NR4A2*^*–*^ (left) and 80,576 *NR4A2*^+^ (middle) nuclei from eight neurotypical donors. Profiles colored green are from clusters identified as DA neurons. **c**, Bar plot of proportions of DA neurons per replicate for *NR4A2*^*–*^ versus *NR4A2*^+^ libraries (median fold enrichment, 70; *n* = 21 *NR4A2*^+^ and *n* = 16 *NR4A2*^*–*^ libraries). **d**, UMAP representation of 15,684 DA neuron nuclei, colored by cell type. **e**, Dot plot showing expression of selected marker genes across DA clusters. **f**, Dendrogram showing phylogenetic relationships among the five species samples in this study. Red and blue branches denote primate and nonprimate species, respectively. **g**, UMAP representation of 6,253 DA neuron nuclei (3,400 primate nuclei and 2,853 nonprimate cells), colored by species (left), cluster identity (middle) and primate versus nonprimate (right). **h**, Stacked bar plot showing the proportion of primate profiles (red bars) in each cluster. **i**, Heatmap of scaled regulon activity, as determined by SCENIC (Methods), for the top three differentially expressed regulons per DA subtype. Bold indicates those TFs previously identified as important for midbrain DA differentiation.

**Extended Data Fig. 1:**
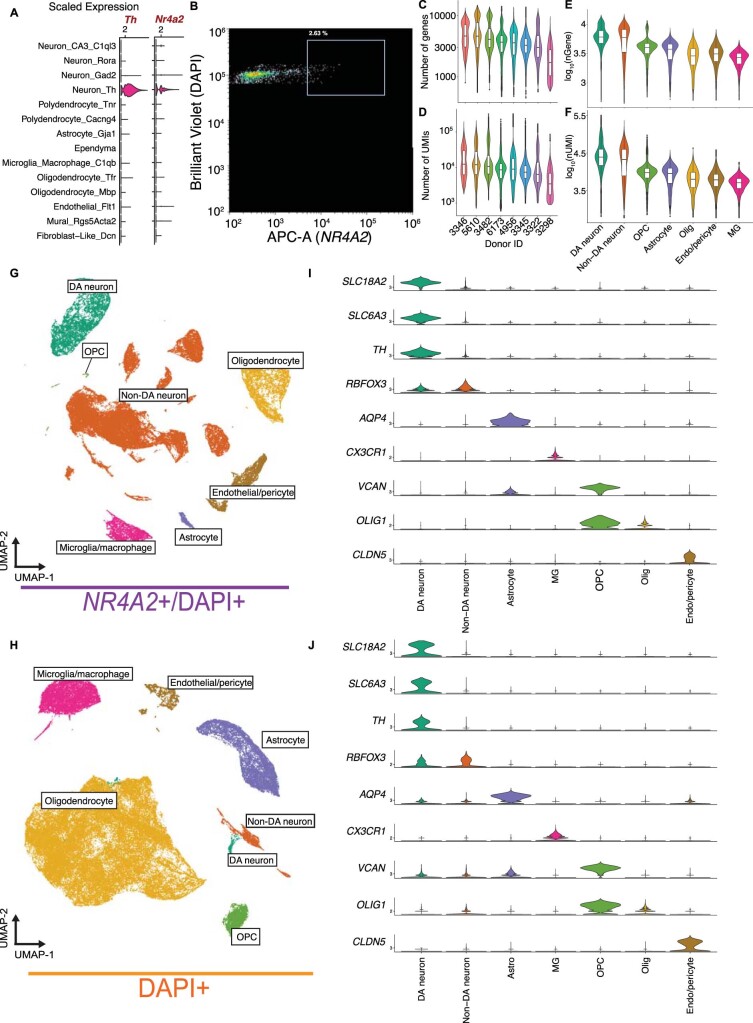
High-throughput snRNA-seq profiling of SNpc. **a**, Expression of tyrosine hydroxylase (*Th*) and *Nr4a2* in a published scRNA-seq study of mouse midbrain(*21*). **b**, Representative FANS plot for enriching midbrain dopaminergic neurons. APC-A (x-axis) represents *NR4A2* antibody channel and Brilliant Violet (y-axis) represents DAPI channel. The *NR4A2* gate was thresholded to select the top 2.5–4% of all nuclei (red box). **c,d**, Number of genes (**C**) and UMIs (unique molecular indicators) (**D**) per nucleus grouped by donor (n = 11,577 nuclei for subject 3298; 49,759 nuclei from 3322; 29,230 nuclei for 3345; 23,747 nuclei for subject 3346; 24,856 nuclei for subject 3482; 25,243 nuclei for subject 4956; 6,967 for subject 5610; and 13,294 nuclei for subject 6173). **e**-**f**, log_10_ number of genes (nGene) (**E**) and log_10_ number of UMIs (nUMI) (**F**) per cell type (n = 15,684 DA neuronal nuclei; 46,860 non-DA neurons; 5,866 OPC nuclei; 14,579 astrocyte nuclei; 76,837 oligodendrocyte nuclei; 16,755 microglia/macrophage nuclei; and 8,092 endothelial cell/fibroblast nuclei). For all box plots, center bar indicates median value and lower and upper hinges correspond to first and third quartiles respectively. Whisker distance from upper and lower hinges represents ≤1.5× !QR. DA, dopaminergic; non-DA, non-dopaminergic, OPC, oligodendrocyte precursor cell; Olig, oligodendrocyte; Endo/pericyte, endothelial cells/pericytes; MG, microglia/macrophage. **g,h**, UMAP representations of the *NR4A2* + (**G**) and *NR4A2-* (**H**) gated nuclei, colored by cluster. **i**,**j**, Violin plots of marker genes for the seven major cell classes in the *NR4A2* + (**I**) and *NR4A2*- (**J**) gated nuclei. DA, dopaminergic; non-DA, non-dopaminergic; Astro, astrocyte; MG, microglia/macrophages; OPC, oligodendrocyte precursor cell; Olig, oligodendrocyte; Endo, endothelial cells.

To address the sampling challenge associated with DA neuron profiling, we developed a protocol based on fluorescence-activated nuclei sorting (FANS), to enrich midbrain DA neuron nuclei for use in snRNA-seq (Fig. 1a). In a scRNA-seq dataset of mouse midbrain^22^ (Extended Data Fig. 1a), we identified the gene *Nr4a2* encoding a transcription factor (TF) as specific to mammalian midbrain DA neurons (area under the curve (AUC) = 0.76; Methods). To comprehensively profile all cell types in the SNpc, we isolated nuclei from eight neurotypical donors (Supplementary Table 1) and performed snRNA-seq on both *NR4A2*-positive and -negative nuclei (Extended Data Fig. 1b). In total, we generated 184,673 high-quality profiles (median number of unique molecular identifiers (UMIs) per individual, 8,810; median number of genes per individual, 3,590 (Extended Data Fig. 1c,d); median number of UMIs per cell, 8,086; median number of genes per cell, 3,462 (Extended Data Fig. 1e,f)), 43.6% of which were from the *NR4A2*-positive cytometry gate (Methods and Fig. 1b). We performed clustering analysis of each donor separately to assign profiles to one of seven main cell classes, including DA neurons (Methods and Extended Data Fig. 1g–j). The *NR4A2*-sorted profiles were 70-fold enriched for DA neurons (Fig. 1c), defined by a cluster with joint expression of *TH*, *SLC6A3* and *SLC18A2* (Extended Data Fig. 1i,j), genes whose products are essential for DA neurotransmission^23^.

**Extended Data Fig. 2:**
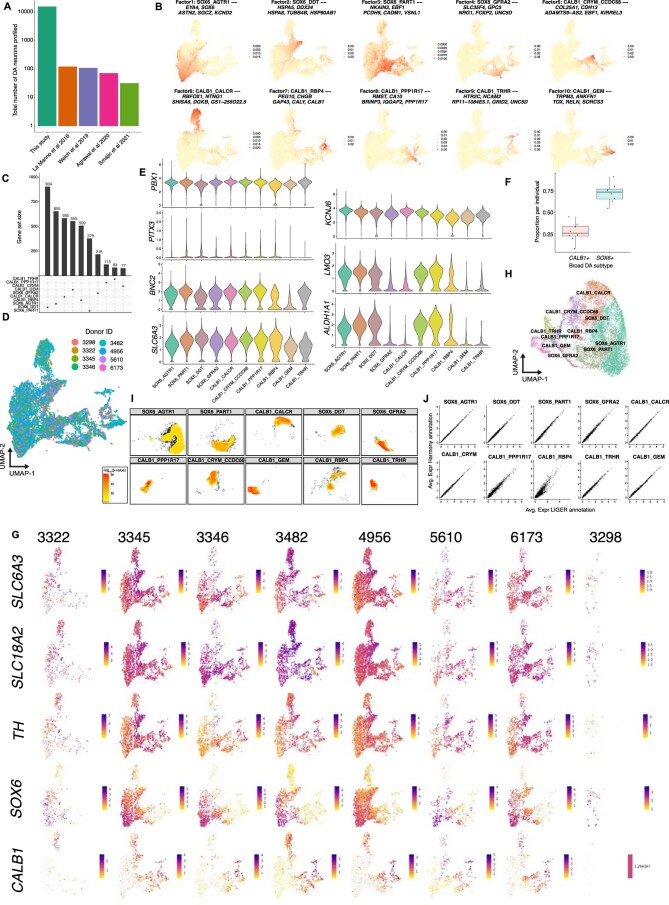
High-throughput profiling and characterization of human midbrain DA neuron diversity. **a**, Bar plot (log scale) of total number of midbrain DA neurons profiled in this study and all previous single-cell studies of the human midbrain. **b**, Factor loadings obtained from LIGER integrative analysis projected onto UMAP embedding of DA neuron analysis. Title of each plot indicates highest loading DA subtype and top five highest loading genes (see Methods) for each factor. **c**, Upset plot of gene sets specific to each DA subtype determined by MAST differential expression analysis (Methods) **d**, SNpc UMAP representation of 15,684 DA profiles colored by individual. **e**, Violin plots showing scaled expression of genes previously defined by La Manno et al. as specific to midbrain DA neurons (left) and specific to subtypes identified in the mouse brain (right) **f**, Box plot of proportion of *SOX6* + and *CALB1* + DA neurons per individual (n = 8 neurotypical controls). Center bar indicates median value and lower and upper hinges correspond to first and third quartiles respectively. Whisker distance from upper and lower hinges represent ≤1.5× IQR. **g**, Expression of (in descending order) *SLC6A3, SLC18A2, TH, SOX6*, and *CALB1* per individual. **h**, UMAP embedding of DA neurons colored by annotations determined via Harmony (see Methods). **i**, Density plots depicting -log10-transformed p-values of a resampling test (see Methods) to determine concordance of LIGER annotations in the Harmony-derived UMAP embedding. **j**, Scatter plot depicting average expression per DA subtype for the LIGER-derived annotations (X-axis, Fig. 1d) and Harmony-derived annotations (Y-axis, Extended Data Fig. 2h).

To identify subtypes of DA neurons, we performed LIGER^17^ on 5,684 high-quality DA neuron nuclei, a 180-fold increase in absolute numbers over existing datasets of human midbrain DA neurons (Extended Data Fig. 2a). Based on our LIGER-derived, low-dimensional embedding (Extended Data Fig. 2b), we identified ten transcriptionally distinct subpopulations (Methods, Fig. 1d,e and Extended Data Fig. 2c) with strong alignment across all donors (Methods; alignment score, 0.98; Extended Data Fig. 2d). Four DA clusters preferentially expressed *SOX6* while the other six expressed *CALB1*, recapitulating a well-defined developmental axis of variation within midbrain DA neurons (Fig. 1e)^24^. All of our subtypes showed strong expression of genes that are essential for DA neurotransmission (Extended Data Fig. 2e)^23^. Previously defined dopaminergic markers^20^ also showed strong expression across our dataset, with mixed expression of subtype markers previously found that differentiate different rodent populations (Extended Data Fig. 2e)^20^. Further, the proportions of these broad subtypes generally matched stereotactic estimates of *CALB1*^+^ cells from previous immunohistochemical stains of postmortem human nigra^21^ (Extended Data Fig. 2f), suggesting no intrinsic bias in our tissue sampling method. The broad *CALB1*–*SOX6* axis of variation was consistent across donors (Extended Data Fig. 2g) and clusters were consistently represented across integrative tools (Extended Data Fig. 2h–j and Methods), suggesting that our strategy consistently uncovered and preserved underlying biological states.

**Extended Data Fig. 3:**
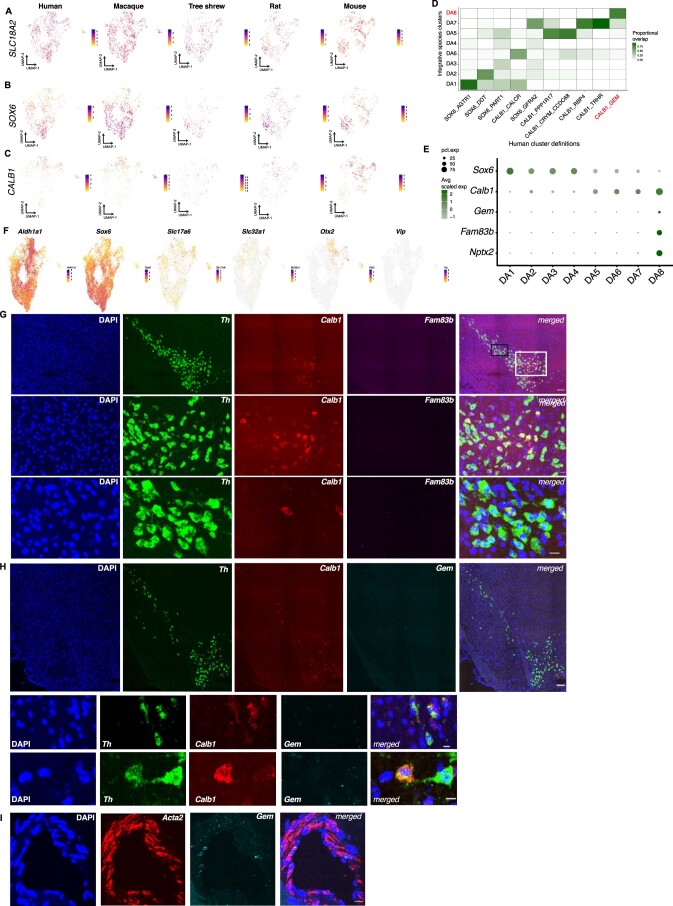
Cross-species analysis of DA subtypes. **a–c**, Expression of *SLC18A2* (DA neuron marker) (**A**), *SOX6* (**B**), and *CALB1* (**C**) across all five species sampled. **d**, Confusion matrix showing overlap of human cells within clusters defined by the cross-species integrative analysis and the human-only analysis (Fig. 1d). The clusters colored in red correspond to the primate-specific population (DA8) and cognate population in the human-only analysis (CALB1_GEM). **e**, Dot plot of additional marker genes for the primate-specific population, DA8. **f**, Feature plots of genes defined by Poulin et al as specific to subtypes of DA neurons in the mouse midbrain. **g**, Single-molecule fluorescence *in situ* hybridization (smFISH) images depicting absence of *Fam83b* + mouse nigral DA neurons. Top row = scanned and tiled image. Middle = white inset from top row indicating midline DA neurons. Bottom row = black inset from top row showing high-resolution images of mouse pars compacta dorsal tier. *Th (*green), *Calb1* (red), and *Fam83b*. Top row scale bar = 100 microns. Middle/bottom row scale bar = 10 microns. **h**, Single-molecule FISH images depicting absence of *Gem* + mouse nigral DA neurons. Top = scanned and tiled view of *in situ* hybridization stains of mouse midbrain. Middle and bottom rows indicate high resolution example images of *Th* + */Calb1* + */Gem-* cells in the dorsal tier (middle) and midline A9 region (bottom). Top row scale bar = 100um. Middle/bottom row scale bar = 10 microns. **i**, High-resolution view of *in situ* hybridization stains of *Acta2* and *Gem* showing detection of *Gem* in smooth muscle vasculature but not DA neurons. Scale bar = 20 microns. Experiment was repeated twice for *Fam83b* and *Gem in situ* analyses and once for the *Acta2* co-localization in mural cells.

Our method for high-throughput profiling of DA subtypes enabled us to make robust comparisons amongst pars compacta DA neurons across species. Although recent profiling studies of primates have shown strong evolutionary conservation of cell types in mice, a few primate-specific specializations have been reported^25,26^. To investigate the evolutionary conservation of our human DA neuron subtypes, we sampled and integrated, with our human datasets, DA neuron profiles from four additional species spanning three phylogenetic orders: Primate, Scandenti and Rodentia (Fig. 1f). Cross-species analysis (Methods) identified eight clusters (Fig. 1g) that consistently followed the *SOX6*–*CALB1* axis of variation (Extended Data Fig. 3a–c). While some profiles exhibited divergence in cellular proportions, integrative analysis largely maintained the cluster distinctions found in the human-only analysis (Extended Data Fig. 3d). Examining the contributions of each species to each cluster, we found that the DA8 population, composed primarily of the CALB1_GEM human population (Extended Data Fig. 3d), included profiles derived only from humans and macaque (Fig. 1g,h), and expressed a number of highly specific marker genes not found in any one other DA subtype in our integrative analysis (Extended Data Fig. 3e) and absent from a previous analysis of rodent DA subtypes (Extended Data Fig. 3f)^23^. Using in situ hybridization across the mouse midbrain (Methods), we found no murine DA neurons expressing the marker genes *Fam83b* and *Gem* for our DA8 population (Extended Data Fig. 3g–i), further confirming the lack of a cognate population in rodents.

### Regulatory element identification in dopaminergic neurons

**Extended Data Fig. 4:**
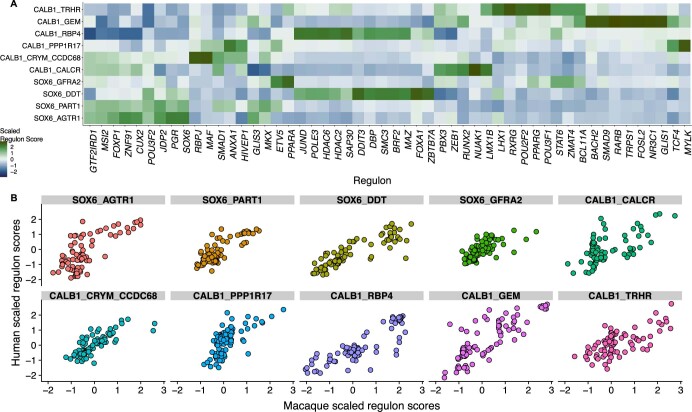
Additional regulon analyses across all DA subtypes. **a**, Top ten differentially enriched (FDR-adjusted p-value < 0.05, Wilcoxon rank-sum two-sided test) regulons per DA subtype. Scaled regulon score is the column-wise average scaled regulon activities per subtype as determined from SCENIC analysis (see Methods). **b**, Scatter plot indicating correlation of between scaled significant regulons from SCENIC analysis of macaque (X-axis) and human (Y-axis) DA subtype profiles.

The identification of regulatory elements that drive the molecular identity of DA neurons can inform differentiation protocols for in vitro studies of DA neurons in PD, as well as drive the refinement of cellular replacement therapies for this disease. To understand the regulatory networks that may drive such transcriptional variation, we used single-cell regulatory network inference and clustering (SCENIC) to identify 84 regulons that were highly specific (*P* adjusted < 0.05 and AUC > 0.7, Wilcoxon rank-sum test; Methods) to the ten DA subtypes defined in our dataset. The top TFs ranked by AUC per DA subtype contained many TFs previously implicated in specifying DA identity, including those encoded by the genes *SOX6*, *OTX2*, *SMAD1*, *PBX1*, *LMX1B*, *NFE2L1* and *EN2* (Fig. 1i and Extended Data Fig. 4a)^27^. Even within the more homogeneous *SOX6* axis we identified several TFs with differential activity across subtypes, including the TF encoded by *SMAD1*, as well as some not previously implicated in DA neuron differentiation such as those encoded by *CUX2* and *ZNF91*. The same SCENIC analysis on DA neuron data generated from the macaque midbrain (Extended Data Fig. 4b) identified highly overlapping sets of regulons, further corroborating the identification of selectively active TFs in each DA subtype.

### Localization of DA neurons in macaque midbrain by Slide-seq

**Fig. 2:**
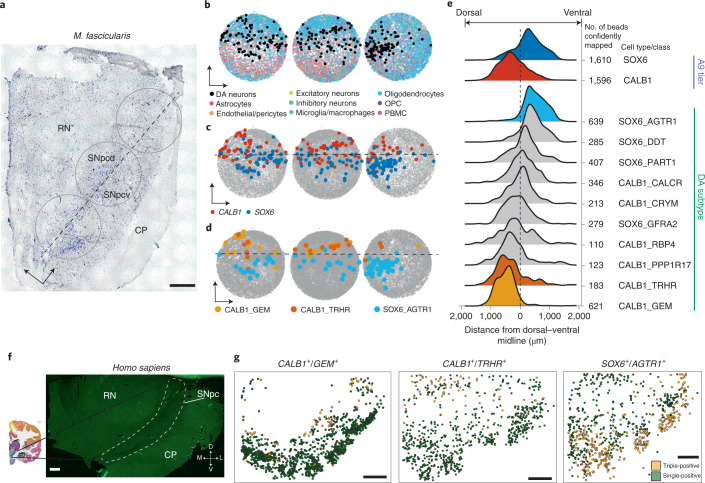
Spatial localization of DA subtypes in NHP and human midbrain. **a**, Nissl staining of a 10-μm *M. fascicularis* midbrain slice adjacent to Slide-seq-assayed tissue. Circles indicate approximate location of the placement of the three Slide-seq arrays shown in **b**–**d**. RN, red nucleus; CP, cerebral peduncles; SN_pcd_, substantia nigra pars compacta dorsal; SN_pcv_, substantia nigra pars compacta ventral. Cartesian arrows indicate orientation of bead arrays in **b*–*d**; scale bar, 1 mm. Nissl staining was repeated nine times across macaque brain. **b**–**d**, Bead arrays colored by RCTD cell type definitions (Methods) corresponding to major cell type (**b**), CALB1^+^ or SOX6^+^ subtypes (**c**) and the three most spatially variable DA subtypes (**d**). **e**, Ridge plot for aggregated densities of CALB1 and SOX6 subtypes (top) and all ten DA subtypes (bottom) across 27 bead arrays (Methods, also includes definition of midline). **f**, Tiled image of one 10-μm human midbrain slice. White dotted line indicates the approximate A9 region; scale bar, 1 mm. Experiment was repeated once. **g**, Scatter plots showing relative location of triple- (yellow) and single-positive cells (Methods) from in situ hybridization of markers: *CALB1*^+^/*GEM*^+^ (left), *CALB1*^+^/*TRHR*^+^ (middle) and *SOX6*^+^/*AGTR1*^+^ DA neurons (right); scale bars, 1 mm. Experiment was repeated five times for *SOX6*^+^/*AGTR1*^+^ localization and once for *CALB1*^+^/*GEM*^+^ and *CALB1*^+^/*TRHR*^+^.

**Extended Data Fig. 5:**
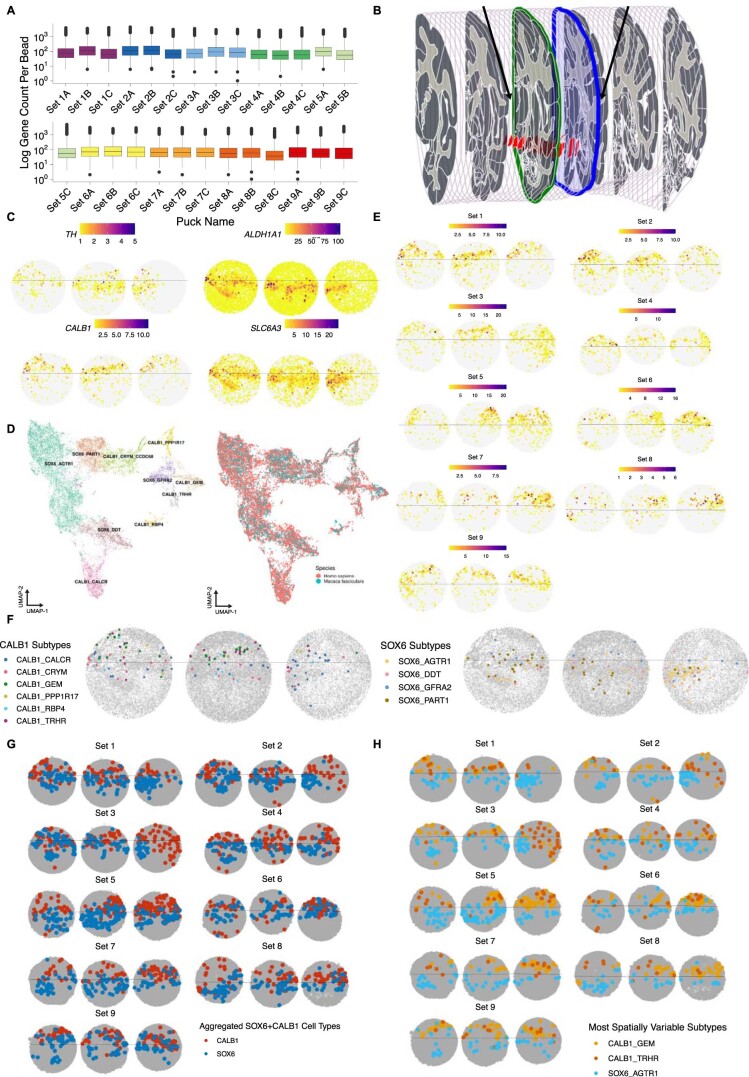
Slide-seq mapping of DA neurons. **a**, Box plots showing, for each bead array, the log number of genes captured per bead (n = 27 bead arrays). Each bead array is a part of a triplet set serially created during the dissection (Methods). Center bar indicates median number of genes and lower and upper hinges correspond to first and third quartiles of data, respectively. Whisker distance from upper and lower hinges represent no more than 1.5*interquartile range. **b**, Anatomical plates of *Macaca fascicularis* brain (*71*) in sagittal view with coronal sectioning. Highlighted red region is the substantia nigra. Black arrows indicate approximate locations of start (green highlighted plate) and end (blue highlighted plate) of sets of bead arrays sampled via Slide-seq. **c**, Spatial plot for the pucks in Set 1 showing each bead’s expression of *TH*, *ALDH1A1*, *CALB1*, and *SLC6A3*. **d**, UMAP showing integrative analysis (Methods) of control DA neuron snRNA-seq profiles and *Macaca fascicularis* DA neuron snRNA-seq profiles. Left — colored by DA types defined by the human integrative analysis (Fig. 1d). Right — colored by species. **e**, Expression of *CALB1* across all 27 bead arrays showing putative location of the substantia nigra pars compacta dorsal tier. **f**, Bead arrays showing localization of all 10 DA subtypes from Set 1 (Fig. 2b-d) as determined by RCTD (Methods). **g**, Location of aggregated SOX6 + and CALB1 + subtypes across all 27 bead arrays. **h**, Location of three highly spatially variable subtypes (SOX6_AGTR1, CALB1_TRHR, CALB1_GEM) across all 27 bead arrays.

**Extended Data Fig. 6:**
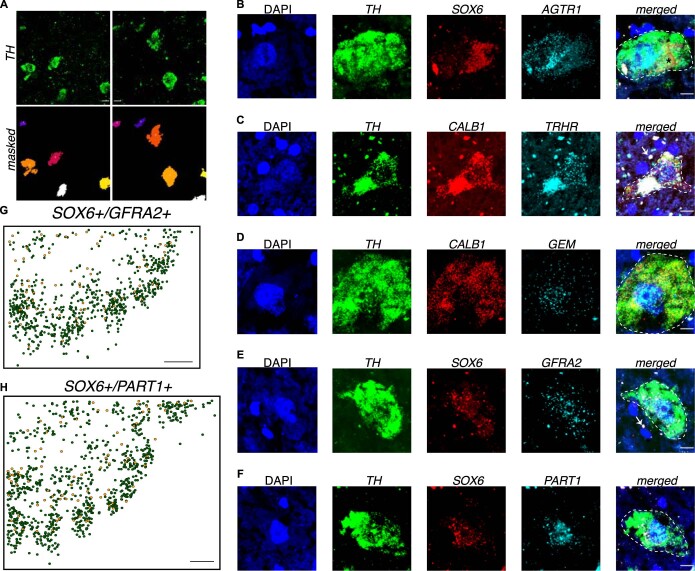
Single-molecule fluorescence *in situ* hybridization of DA neurons in postmortem human midbrain. **a**, Top row - representative images of *TH in situ* hybridization in postmortem midbrain (Methods, scale bar = 10um). Bottom row - masks obtained from FIJI denoting location and size of DA neuron (Methods). **b–f**, Representative images for: *TH* + */SOX6* + */AGTR1* + (**B**), *TH* + */CALB1* + */TRHR* + (**C**), *TH* + */CALB1* + */GEM* + (**D**),*TH* + */SOX6* + */GFRA2* + (**E**), *TH* + */SOX6* + */PART1* + (**F**). White arrows indicates lipofuscin-associated autofluorescence and * indicates neuromelanin-associated autofluorescence defined by the absolute co-localization of pixel values across all channels. Scale bar = 10um. **g,h**, Location of *SOX6* + */GFRA2* + (**G**) and *SOX6* + */PART1* + (**H**) across human midbrain, yellow = triple-positive cells, green = single-positive (*TH* + ) cells. Sections for (**G**) and (**H**) were obtained from 1.8 mm from the most rostral/anterior aspect of the pars compacta. Scale bar = 1 mm for (**G**) and (**H**). Experiment was repeated once for each subpopulation.

Dopaminergic neurons in the SNpc comprise the A9 group of catecholaminergic neurons and are divided into dorsal and ventral tiers, with the ventral tier showing greater vulnerability to PD-associated degeneration^6,21,28–30^. To spatially localize the ten DA populations defined in our human analysis (Fig. 1d), we performed Slide-seq^31,32^ on 27 arrays generated from nine coronal sections spanning the caudal 80% of the rostralcaudal axis of a *Macaca fascicularis* SNpc (Methods, Fig. 2a and Extended Data Fig. 5a,b). The A9 group was easily identifiable by visualizing the expression of DA neuron markers *TH* and *SLC6A3* (Extended Data Fig. 5c). Using a recently described tool for decomposition of cell type mixtures in spatial transcriptomics data^33^ (Methods), we localized our ten DA subtypes to 3,206 Slide-seq-defined bead locations in the A9 group (Fig. 2d, Extended Data Fig. 5d and Methods) and positioned each relative to the demarcation between dorsal and ventral tiers (Fig. 2e, dotted line), defined from the expression of *CALB1* and *ALDH1A1* (Extended Data Fig. 5c–e)^21^. The SOX6_AGTR1 population was the most strongly enriched in the ventral tier (Fig. 2d,e and Extended Data Fig. 5f–h; mean SOX6_AGTR1 relative distance, +490 μm), consistent with previous marker analyses performed on laser-capture microdissection subsets of A9 DA neurons^34^, while the CALB1_GEM and CALB1_TRHR populations were strongly enriched in the dorsal tier (Fig. 2d,e and Extended Data Fig. 5f–h; mean CALB1_GEM relative distance, −478 μm; mean CALB1_TRHR relative distance, −288 μm). Single-molecule fluorescence in situ hybridization (smFISH) (Extended Data Fig. 6a and Methods) of the human midbrain confirmed localization of the SOX6_AGTR1 subtype to the ventral tier (Fig. 2f,g and Extended Data Fig. 6b) and types CALB1_GEM and CALB1_TRHR to the dorsal tier (Fig. 2f,g and Extended Data Fig. 6c,d). Additional in situ analyses of two other *SOX6*_+_ populations confirmed their lack of selective localization to either tier (Extended Data Fig. 6e–h), consistent with the Slide-seq results (Fig. 2e).

### Differentially vulnerable DA neurons in PD

**Extended Data Fig. 7:**
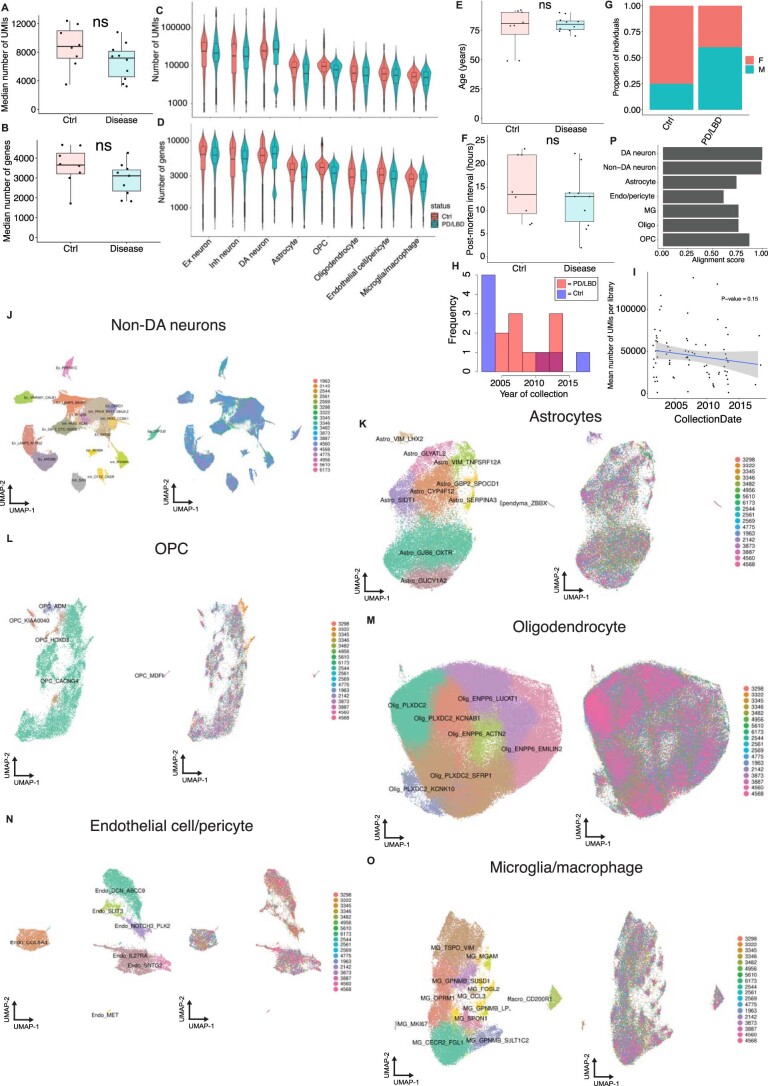
Case-control scRNA-seq integrative analysis of SNpc. **a,b**, Box plots showing, per individual, the median number of UMIs (**A**) and genes (**B**) per profile (n = 8 neurotypical controls and 10 PD/LBD samples). ns, not significant Wilcoxon rank-sum two-sided test p > 0.05. Center bar indicates median value and lower and upper hinges correspond to first and third quartiles respectively. Whisker distance from upper and lower hinges represent no more than 1.5*interquartile range. **c**,**d**, Violin plots of number of UMIs (**C**) and genes (**D**) per profile across eight major cell classes grouped by disease (PD/LBD, blue) and control (Ctrl, red). **e-f**, Box plots showing, per individual, the age at death (**E**) and postmortem interval (**F**) stratified by case (PD/LBD) and control (Ctrl) (n = 8 neurotypical controls and 10 PD/LBD samples). Center bar indicates median value and lower and upper hinges correspond to first and third quartiles respectively. Whisker distance from upper and lower hinges represent no more than 1.5*interquartile range (ns = not significant, Welch two sample t-test, p = 0.61 for (**E**) and p = 0.18 for (**F**). **g**, Stacked bar plot of sex stratified by disease status. **h**, Histogram of the year of collection of postmortem midbrain samples colored by case-control status (see Supplementary Tables 1 and 2 for raw values). **i**, Scatter plot of the collection year for each sample (x-axis) and the median number of UMIs (y-axis). Blue line indicates line of best fit as determined by lm.fit in R. Grey shading indicates standard error of fit. P-value corresponds to significance of slope from a mixed-effect model (see Methods). **j–o**, UMAP representations of Non-DA neurons (n = 91,479 nuclei) **(J)**, Astrocytes (n = 33,506 nuclei) **(K)**, Oligodendrocyte precursor cells (OPCs) (n = 13,691 nuclei) **(L)**, Oligodendrocytes (n = 178,815 nuclei) **(M)**, Endothelial cells/pericytes (n = 14,903 nuclei) **(N)**, and Microglia/macrophages (n = 33,041 nuclei) **(O)** colored by cell type (left) and individual (right). **P**, Alignment scores (see Methods) for each of the seven non-DA cell classes.

The strong ventral localization of the SOX6_AGTR1 population suggested that it may be especially susceptible to PD-associated degeneration. To identify cell-type-specific molecular alterations in PD, we profiled an additional 202,810 high-quality nuclei (Extended Data Fig. 7a–d; median number of UMIs per individual, 7,177; median number of genes per individual, 3,108; median number of UMIs and genes per cell, 6,939 and 3,076, respectively), including 6,364 DA neurons, from ten age-matched and postmortem-interval-matched (Extended Data Fig. 7e–g) individuals with documented pathological midbrain DA neuron loss, and a clinical diagnosis of either PD or LBD (Supplementary Table 2). Between PD/LBD and neurotypical control tissues there were no significant differences in tissue collection date (Extended Data Fig. 7h), and this covariate had no significant effect on the integrity of capture (*P* > 0.05; Methods and Extended Data Fig. 7i). Finally, integrative analysis of these donors with our neurotypical controls (Methods) identified 68 transcriptionally defined subpopulations (Extended Data Fig. 7j–o) from our seven major cell classes, with minimal batch-dependent variation (alignment scores: 0.61–1.0; median alignment score across cell types, 0.76; Extended Data Fig. 7p).

**Fig. 3:**
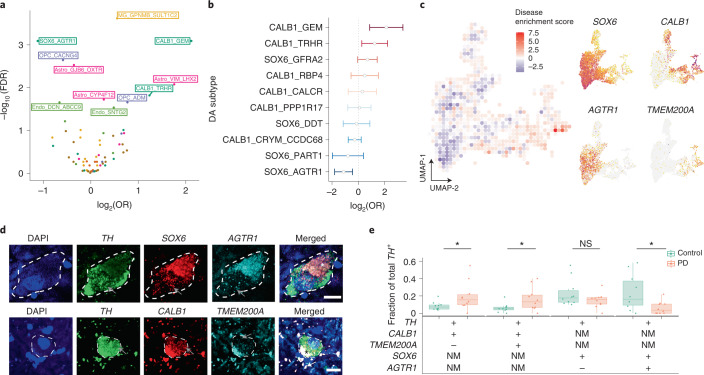
Quantification of DA subtype vulnerability to PD-associated degeneration. **a**, Volcano plot showing OR and FDR computed by MASC (Methods) for each of the 68 clusters identified in SNpc snRNA-seq analysis. Labeled clusters are those significantly (FDR-adjusted *P* < 0.05) increased or depleted in association with PD/LBD. Points and text are colored by major cell type: dark green, DA neurons; yellow, microglia/macrophages; purple, OPCs; light green, endothelial cells/pericytes; pink, astrocytes. **b**, OR estimate of ten dopaminergic subpopulations as identified by MASC. Center of bar corresponds to OR estimate obtained from MASC, width corresponds to 2.5× s.d. of OR estimate from MASC. Bars that cross zero (dotted line) not statistically significant (FDR-adjusted *P* > 0.05, *n* = 22,048 DA neurons sampled across ten PD/LBD donors and eight neurotypical donors). **c**, Left**:** disease enrichment score (Methods) overlaid onto a binned UMAP representation of integrative analysis of both PD/LBD and control DA neurons (*n* = 10 PD/LBD individuals and *n* = 8 neurotypical controls). Right: expression of selected genes used to validate subtype vulnerability plotted on UMAP representation of DA neurons. **d**, Representative images of triple-positive cells for a disease-resistant DA population (*TH*^+^/*CALB1*^+^/*TMEM200A*^+^) and a disease-vulnerable population (*TH*^+^/*AGTR1*^+^/*SOX6*^+^, bottom). White/black asterisks indicate neuromelanin-induced autofluorescence while white arrows show lipofuscin-induced autofluorescence; gray arrows indicate RNA puncta. Scale bars, 10 μm. **e**, Boxplot showing proportions of four DA populations across ten PD and ten control SNpc tissue donors, determined by counting smFISH images from the two staining procedures (3,258 and 2,081 DA neurons counted for first and second assay, respectively) described in **d**. Center line of the boxplot indicates the median value while upper and lower hinges indicate the first and third quartiles of data, respectively. Whisker distance between upper and lower hinges represent ≤1.5× interquartile range. All dots represent an individual case for each subtype as a fraction of total *TH*^+^ cells counted. +, positive for marker; −, negative for marker; NM, not measured; NS, not significant. *P < 0.05 (*P* = 0.041 for *CALB1*^+^/*TMEM200A*^+^/*TH*^+^ comparison, *P* = 0.028 for *CALB1*^+^/*TMEM200A*^−^/*TH*^+^ comparison, *P* = 0.009 for *CALB1*^+^/*TH*^+^ comparison, *P* = 0.024 for *SOX6*^+^/*AGTR1*^+^/*TH*^+^ comparison, *P* = 0.28 for *SOX6*^+^/*AGTR1*^*–*^/*TH*^+^ experiment and *P* = 0.015 for *SOX6*^+^/*AGTR1*^*−*^/*TH*^+^ comparison; Wilcoxon rank-sum two-sided test; Methods).

**Extended Data Fig. 8:**
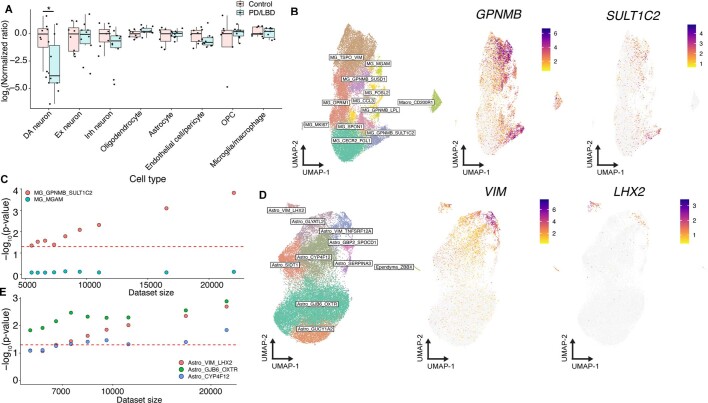
Analyses and robustness testing of cell type proportional changes in PD/LBD. **a**, Box plot of log_2_ ratio of cell types normalized to median of control ratios. Pink and blue dots denote control and PD/LBD individuals, respectively (* = p < 0.05, p = 0.034, Wilcoxon rank-sum two-sided test, n = 8 neurotypical controls and 10 PD/LBD postmortem samples). Center bar indicates median value and lower and upper hinges correspond to first and third quartiles respectively. Whisker distance from upper and lower hinges represent no more than 1.5*interquartile range. **b**, Left = UMAP of 33,041 microglia/macrophage cells. Middle and right = expression of *GPNMB* and *SULT1C2* marking specific expression in the annotated microglia cluster. **c**, Dot plot of downsampling analysis for two microglia subtypes. Red dotted line is FDR-adjusted p-value = 0.05. **d**, Left = UMAP of 33,506 astrocytes. Middle and right = expression of *VIM* and *LHX2* respectively marking specific expression in the annotated astrocyte cluster. **e**, Dot plot of downsampling analysis for three astrocyte subtypes. Red dotted line is FDR-adjusted p-value = 0.05.

We assessed the differential abundance between PD/LBD and aged control samples for both major cell classes and all molecularly defined subtypes. Among major cell classes, DA neurons showed the largest significant decline (*P* < 0.05, Wilcoxon rank-sum test) as a fraction of cells per individual (Extended Data Fig. 8a). Among the 68 molecularly defined subpopulations, 11 showed significant proportional changes in association with PD/LBD (Fig. 3a; false discovery rate (FDR)-adjusted *P* < 0.05, absolute log_2_(odds ratio (OR_) > 0; Methods). One proportionally increased population was a subset of microglia expressing *GPNMB* (Extended Data Fig. 8b), which has been identified as a marker of disease-associated microglia in transcriptomic studies of Alzheimer’s disease (AD)^35,36^ and was robust up to one-sixth of the original dataset size (Extended Data Fig. 8c). We also identified an increase in the VIM_LHX2 astrocyte subtype (Extended Data Fig. 8d), similarly robust to large losses in power due to sample size (Extended Data Fig. 8e). The VIM_LHX2 population expresses reactive markers, namely *VIM* and *LHX2* (Extended Data Fig. 8d), suggesting that this population may play a role in responding to degenerative changes in PD/LBD SNpc.

The largest statistically significant decline was in the SOX6_AGTR1 DA population, while clusters CALB1_GEM and CALB1_TRHR were proportionally increased (Fig. 3b). These proportional changes were robust to differences in absolute numbers of DA neurons sampled per cluster (Supplementary Fig. 1a), clinical diagnosis (Supplementary Fig. 1b) and library quality (Supplementary Fig. 1c,d). We further developed a metric to visualize disease-associated enrichment or depletion within the low-dimensional embedding of jointly analyzed cell profiles, identifying a gradient of susceptibility (Fig. 3c and Methods) that correlated with the expression of *AGTR1* and ORs from mixed-effects association of single cells (MASC) (Fig. 3c).

Our flow cytometry procedure to isolate DA nuclei relies on protein expression of *NR4A2*, which is downregulated in DA neurons in PD^37^. To address these and other potential confounders, we quantified DA subtype proportions using smFISH, performed on an additional 20 postmortem-frozen midbrains equally divided among neurotypical donors and individuals who had died of PD (with concurrent documented midbrain DA neuron loss) (Supplementary Table 3, Supplementary Fig. 2a–d and Methods). In the first experiment we probed for cells expressing *TH*, *SOX6* and *AGTR1* (Fig. 3d); in a second experiment, we identified cells in situ expressing *TH*, *CALB1* and *TMEM200A* (Fig. 3d), a marker enriched in the CALB1_TRHR, CALB1_GEM and CALB_RBP4 populations (Fig. 1e). We assayed the proportional representations of double- and triple-positive cells in each smFISH experiment and counted a total of 5,339 individual DA neurons (Supplementary Fig. 2e) across 40 full SNpc sections. We confirmed that *CALB1*^+^/T*MEM200A*^+^ DA cells were selectively enriched in PD (Fig. 3e; Wilcoxon rank-sum test *P* < 0.05), as were *CALB1*^+^/*TMEM200A*^*−*^ DA cells, although the log fold change difference was lower (Fig. 3e; log_2_(fold change 1.13) for the *TMEM200A*^*−*^ group versus log_2_(fold change 1.32) for the *TMEM200A*^+^ group; Supplementary Table 5). We also confirmed selective depletion of the SOX6_AGTR1 subpopulation (Fig. 3e; Wilcoxon rank-sum test *P* < 0.05, log_2_(fold change −2.1); Supplementary Table 5).

### Cellular localization of PD common variants

**Fig. 4:**
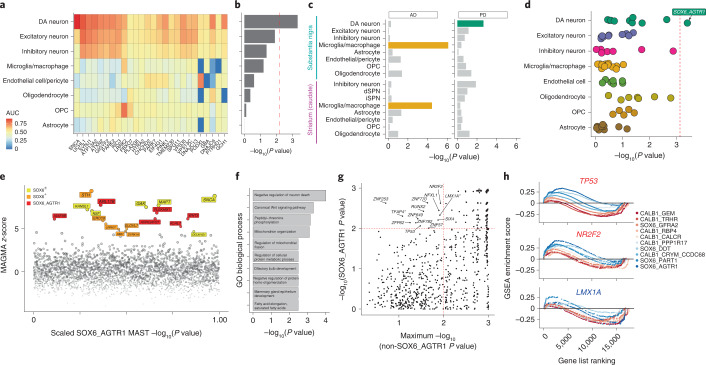
Genetic enrichments and TF set analyses within DA subtypes. **a**, Heatmap of expression of 26 familial genes, colored by AUC statistic (Presto; Methods). **b**, Bar plot of −log_10_-transformed *P* values from Fisher’s exact test comparing overlap between markers for major cell types (Methods) and familial variant genes. Red dashed line indicates Bonferroni significance threshold (*P* < 0.05) **c**, Bar plot of −log_10_-transformed *P* values from MAGMA enrichment of AD (left) and PD (right) across 16 cell types from dorsal striatum (caudate) and SNpc. Bars are colored for significantly (Bonferroni-corrected *P* < 0.05) enriched cell types. **d**, Dot plot of −log_10_-transformed *P* values for MAGMA analysis of PD genetic risk in 68 transcriptionally defined SNpc clusters. Clusters are grouped on the *y* axis by major cell class. Red dashed line indicates Bonferroni significance threshold (*P* < 0.05). **e**, Scatter plot of genes nominated from MAGMA gene-based analysis (*y* axis) and enriched in the SOX6_AGTR1 set (*x* axis). Red indicates genes not differentially expressed in any other DA subtype; orange indicates genes enriched within all SOX6^+^ DA subtypes; yellow indicates genes enriched in SOX6^+^ or SOX6^–^ DA subtype. #, Loci that have the nominated SNP within a coding region of the nominated gene. **f**, GO terms enriched among genes differentially expressed within the SOX6^+^ subtype that also have high (*z*-score > 4.568) MAGMA *z*-scores derived from PD GWA study. **g**, GSEA of TF target sets (Methods) within the SOX6_AGTR1 type (*y* axis) versus the maximum value of any other DA type (*x* axis). Dashed red lines indicate FDR-significant TFs. *, TFs with a negative enrichment score. **h**, GSEA trace plots for three TFs: *TP53*, *NR2F2* and *LMX1A*. Colors correspond to ORs derived from MASC analysis (Fig. 2b). dSPN, direct spiny projection neuron; iSPN, indirect spiny projection neuron.

We next sought to better understand the origins of the selective vulnerability of SOX6_AGTR1 cells to neurodegeneration in PD. One possibility is that the human genetic risk for PD—which is established at birth—selectively acts within the vulnerable population. To test this, we examined the enrichment of expression of genes harboring either familial and common variants associated with PD. A total of 26 genes have been identified that harbor mutations, as ascertained by family studies, that confer substantial risk for PD^38^. We tested the overlap between these genes and markers specifically expressed in eight major cell classes of the SNpc (Fig. 4a and Methods). The DA neuron gene set was the only one to show significant enrichment (Bonferroni-corrected *P* < 0.05, Fisher’s exact test; Methods) of genes that contained these familial variants (Fig. 4b), suggesting that a substantial fraction of these genes act within DA neurons to influence neurodegeneration.

**Extended Data Fig. 9:**
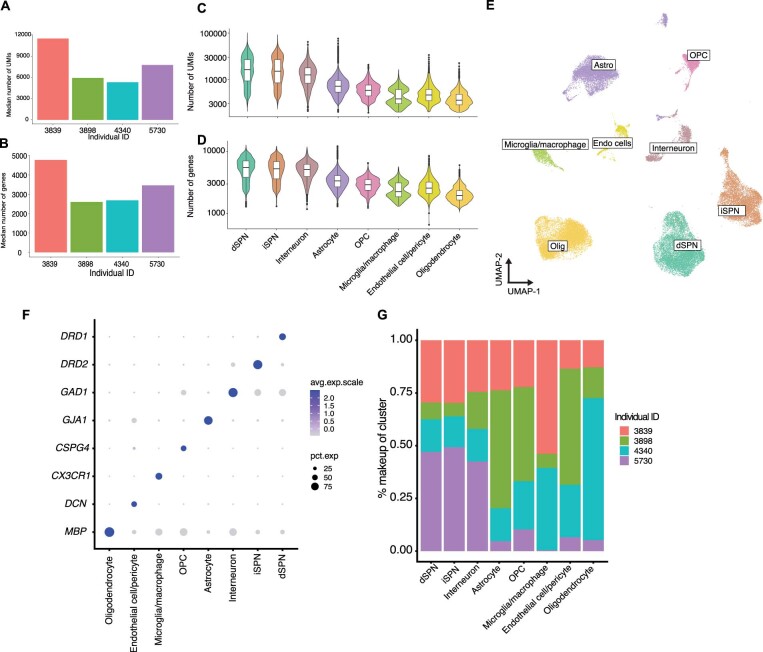
Analysis of human dorsal striatum by snRNA-seq. **a,b**, Median number of UMIs (**A**) and genes (**B**) per donor. **c**,**d** Violin plots showing the number of UMIs (**C**) and (**D**) across eight major cell classes defined by clustering. (n = 9,096 iSPNs; 10,503 dSPNs; 8,433 astrocytes; 1,418 endothelial cells/fibroblasts; 2,115 inhibitory neurons; 2,105 microglia/macrophages; 2,445 OPCs; 10,667 oligodendrocytes), direct spiny projection neurons; iSPN, indirect spiny projection neuron; OPC, oligodendrocyte precursor cells. For box plots in (C) and (**D**), center bars indicates median value and lower and upper hinges correspond to first and third quartiles respectively. Whisker distance from upper and lower hinges represent no more than 1.5*interquartile range. **e**, UMAP representation of 46,872 single nuclei from the dorsal striatum colored by major cell class. Astro, astrocyte; Olig, oligodendrocyte; Endo cells, endothelial cell/pericyte. **f**, Dot plot showing specific expression of selected marker genes across the eight major cell classes. **g**, Percent contribution of four tissue donors to the eight major cell classes.

**Extended Data Fig. 10:**
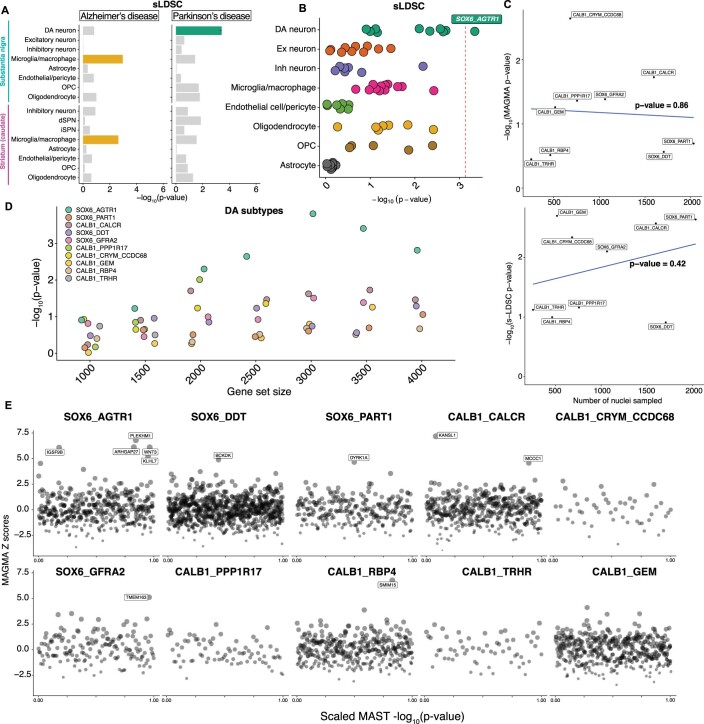
Genetic enrichment analyses of the substantia nigra cell types and subtypes. **a**, Bar plot of -log_10_-transformed p-values from s-LDSC (stratified LD score) enrichment of Alzheimer’s (left) and Parkinson’s disease (right) across 16 cell types from dorsal striatum (caudate) and SNpc. Bars are colored for significantly (Bonferroni-corrected p-value < 0.05) enriched cell types. **b**, Dot plot of -log_10_-transformed p-values for s-LDSC analysis of PD genetic risk in the 68 transcriptionally defined SNpc clusters. Clusters are grouped on the y-axis by major cell class. Red dotted line indicates the Bonferroni significance threshold (p-adjusted < 0.05). **c**, Scatter plot of number of nuclei sampled per DA subtype (x-axis) and -log_10_-transformed p-values from MAGMA (top) and s-LDSC (bottom) analyses. Blue line indicates the line of best fit as determined by lm.fit package (Methods). P-value determined from a two-sided significance testing of the slope coefficient from the best fit line (n = 9 DA subtypes, see Methods). **d**, Dot plot of -log_10_-transformed p-values for MAGMA analysis of DA subtype markers across gene set sizes (Methods) for PD common variant study(*38*). **e**, Pseudo-Manhattan of genes specific to each DA subtype (Extended Data Fig. 2c) ordered by scaled -log_10_-transformed p-values from MAST (Methods). Genes are those prioritized by MAST (Z-score > 4.568).

We next examined the enrichment of common variant risk of sporadic PD within markers of our SNpc cell classes (Methods)^39,40^, as well as markers of cell classes defined by the additional profiling of 46,872 nuclei from postmortem dorsal striatum tissue of four neuropathologically normal individuals (Extended Data Fig. 9a–g and Supplementary Table 4). We observed strong, significant enrichment (Bonferroni-corrected *P* < 0.05) of heritable risk for sporadic PD (Methods) in markers of DA neurons (Fig. 4c, Extended Data Fig. 10a and Supplementary Table 9), in agreement with recent analyses of mouse and human single-cell datasets^19,41^. In contrast, significant enrichment (Bonferroni-corrected *P* < 0.05; Supplementary Table 9) of heritable risk for sporadic AD was identified in markers of microglia/macrophage clusters from both SNpc and dorsal striatum (Fig. 4c and Extended Data Fig. 10a), also consistent with previous analyses^41,42^. Next, we tested the enrichment of PD common variant risk across our 68 transcriptionally defined subpopulations in human SNpc (Methods and Supplementary Table 7). Using two different statistical methods^40,43^, we found the largest and only statistically significant (Bonferroni-corrected *P* < 0.05; Methods and Supplementary Table 9) enrichment of PD genetic risk genes within the SOX6_AGTR1 cell subtype (Fig. 4d and Extended Data Fig. 10b). Relative enrichment by both methods was uninfluenced by the number of nuclei sampled per DA subtype (Extended Data Fig. 10c and Supplementary Table 7), and ordering of the significance of these enrichments was consistent across variations in gene set size (Extended Data Fig. 10d).

To further characterize the genetic loci enriched in SOX6_AGTR1 cells, we identified genes that were assigned high MAGMA *z*-scores and low *P* values for DA subtype differential expression, including the SOX6_AGTR1 population (Fig. 4e and Extended Data Fig. 10e). Some prioritized—and well-studied—genes were expressed in multiple *SOX6*^+^ and *CALB1*^+^ subtypes, including *SNCA*, *MAPT* and *GAK*. Other SOX6_AGTR1-specific genes included the GWA-prioritized genes *WNT3* and *IGSF9B*^39^. Gene Ontology (GO) enrichment analysis (Methods) of SOX6^+^-specific genes identified relevant ontologies that included regulation of neuron death and Wnt signaling^44,45^, reinforcing that the genetic loci identified as being expressed in this subtype probably act cell intrinsically to influence neurodegeneration (Fig. 4f).

### Nomination of gene programs altered in dopaminergic neurons

Finally, we sought to nominate gene regulatory programs significantly and specifically altered in the SOX6_AGTR1 population in our PD/LBD tissue donors. Using gene set enrichment analysis (GSEA)^46,47^ of known TF target sets^48^ (Methods), we identified a total of 13 TFs whose targets were significantly (FDR significant *P* < 0.05) enriched in the SOX6_AGTR1 population but not in the other DA subtypes (Fig. 4g,h and Supplementary Fig. 3a). This analysis revealed a depletion of targets of the TF encoded by the gene *LMX1A* (Methods; normalized enrichment score <0), a TF involved in the developmental specification of midbrain DA neurons^49^. Transcription factors whose targets were enriched in the SOX6_AGTR1 population included *TP53*, whose own transcript was also upregulated specifically in the SOX6_AGTR1 population (Supplementary Fig. 3b), as well as *NR2F2*; both TFs modulate the progression of midbrain DA neuron loss in mouse models of PD^50–52^.

## Discussion

In this study we generated a molecular taxonomy of human SNpc DA neurons, spatially localized them within the SNpc and identified one DA subpopulation, SOX6_AGTR1, that is highly susceptible to neurodegeneration in PD. Importantly, across both the substantia nigra and caudate, this population was the most strongly enriched for expression of genes associated with PD by previous GWAS, suggesting that the genetic risk of PD acts preferentially in the most vulnerable neurons to influence their survival. Transcriptional changes within SOX_AGTR1 cells in patients with PD implicated several canonical cell stress pathways—including those regulated by TFs encoded by the genes *TP53* and *NR2F2*—as important to the process of PD-associated neuronal death.

Our snRNA-seq analysis of SNpc DA neurons provides a comprehensive taxonomy of these critically important cells in humans. Our map will help guide bulk transcriptomic studies of PD in localization of disease-associated signals to specific human DA subtypes. Further, DA subtype definitions will allow the refinement of in vitro DA neuron differentiation protocols, which could prove useful in genetic screens of neuronal susceptibility^53,54^ and the testing of candidate therapeutic molecules. Interestingly, although nine of our ten populations showed homology to rodent DA populations, one cluster of cells, CALB1_GEM, was found only in our snRNA-seq data from macaque and human and not from mouse, rat or tree shrew. We localized CALB1_GEM cells exclusively to the dorsal tier of the SNpc, which is known to be expanded in primates relative to rodents^26,27^. Indeed, primate dorsal tier neurons have previously been shown to make atypical projections directly to cortex^26,27^. The possibility that the molecularly distinct CALB1_GEM population is responsible for these projections is intriguing, but will need to be verified directly in nonhuman primate (NHP) models.

The partitioning of heritable disease risk preferentially to the most vulnerable DA population provides evidence that the genetic influences of PD-associated degeneration are preferentially cell intrinsic. This result—which is consistent with previous efforts to partition heritable risk of PD^19,41^—contrasts with similar analyses performed on late-onset AD genetic risk that particularly implicate microglia and other populations of myeloid origin in nonautonomous neuroimmune mechanisms^55^. Thus, despite overlapping pathologies and the clinical phenomenon of these two diseases, there exist substantial differences in how genetic risk may manifest in producing the disease, an insight that could prove useful in the advancement of specific therapeutic opportunities and biomarkers.

The heritability enrichment within PD-vulnerable neurons themselves also provides a crucial opportunity to address a longstanding question in PD about the primacy of midbrain DA neuron death relative to neurodegeneration in other regions. For example, one hypothesis of PD pathogenesis posits that PD pathology ascends through the brain (for example, via alpha-synuclein aggregates) from outside the central nervous system, through medullary brainstem structures^56^. The extension of profiling efforts to these other structures^57^—and comparison of heritability enrichment in vulnerable cell types—could help to clarify whether these other, vulnerable structures are degenerating due to primary influences of the disease or are secondary to midbrain DA loss.

Our identification of TFs whose activity is up- or downregulated, as nominated by our enrichment of targets in our differential expression, specifically within the vulnerable SOX6_AGTR1 population implicates specific cellular pathways in the process of DA neuron death in PD. The TF encoded by *NR2F2*, for example, has previously been shown to promote mitochondrial dysfunction in several disease models, including those of heart failure^58^ and PD^52^. Upregulation of the TF encoded by *TP53* provides a link to other neurodegenerative diseases, such as amyotrophic lateral sclerosis, in which *TP53* has been implicated in motor neuron death^59–62^. Cross-disorder integrative analyses may reveal conserved molecular processes that are prime candidates for therapeutic intervention.

## Methods

### Ethics statement

All housing and procedures involving rodents were conducted in accordance with the US National Institutes of Health Guide for the Care and Use of Laboratory Animals, under protocol no. 0129-09-16, and were approved by the Broad Institute Committee on Animal Care (IACUC). All NHP tissue was processed in compliance with the Broad Institute IBC (IBC, no. 2016-00127). All human tissue falls under a ‘Not Engage’ designation determined by the Broad Institute IACUC (Federal-wide assurance, no. FWA00014055).

### Animal husbandry of *Mus musculus*

Animals were group housed with a 12/12-h light/dark schedule and allowed to acclimate to their housing environment for 2 weeks post arrival. For *M. musculus* housing, ambient temperature was strictly maintained between 68 and 72 °Fahrenheit and humidity strictly maintained between 30 and 50%. All procedures involving animals at Massachusetts Institute of Technology (MIT) were conducted in accordance with the US National Institutes of Health Guide for the Care and Use of Laboratory Animals, and were approved by the MIT Committee on Animal Care. All procedures involving animals at the Broad Institute were conducted in accordance with the US National Institutes of Health Guide for the Care and Use of Laboratory Animals, under protocol no. 0120-09-16.

### Cryosectioning and brain preparation

Postmortem human midbrain and dorsal striatum (caudate nucleus) tissue were flash-frozen at −80 °C and cryosectioned at −15 to −20 °C into 60-µm sections. For midbrain preparation, pigmented regions of human midbrain were microdissected and between five and ten 60-µm sections prepared. Following microdissection, samples were placed on dry ice.

### Generation of single-nucleus suspensions from frozen human midbrain and caudate samples

To each cryosectioned sample, 1 ml of extraction buffer (ExB) was added to an Eppendorf tube. Samples were triturated before being placed in a six-well plate. Samples were then triturated 20 times with ExB, every 2 min, until no large chunks of tissue were observed. After the last trituration, samples were diluted with 45 ml of wash buffer (WB) in a 50-ml Falcon tube and then split into four 13-ml solutions in 50-ml Falcon tubes. Samples were then spun at 500*g* for 10 min at 4 °C in a swing-bucket benchtop centrifuge.

After centrifugation, samples were placed in an ice bucket and the supernatant aspirated until 50–100 µl of liquid remained.

The pellets were then resuspended in 250 µl of WB and mixed thoroughly by trituration. This 250-µl solution was then pooled with four other pellets from the same original midbrain and resuspended into approximately 1 ml of WB in a 1.5-ml Eppendorf tube.

### Immunolabeling and blocking of human nuclei

Approximately 100 µl of 10% bovine serum albumin in WB (final concentration, 1%) was added to the concentrated nuclei. The antibody NR4A2-A647 (Santa Cruz, no. sc-376984 AF647) was then added at a concentration of 1:350. Additionally, one caudate library was stained and sorted with NeuN (see metadata at Broad SCP link in Data Availability), which was performed by the addition of an anti-NeuN-PE antibody (EMD MilliPore Corp., clone A60; no. FCMAB317PE) at a concentration of 1:1,500. Samples were then covered in aluminum foil and incubated on a rotator at 4 °C for 1 h. Following incubation, Eppendorf tubes were spun at 150*g* for 10 min in a swing-bucket benchtop ultracentrifuge. The supernatant was then gently aspirated, then WB added to achieve a total sample volume of 1 ml. Samples were then stained with DAPI (ThermoFisher, no. 62248) at 1:1,000 dilution and filtered with a 70-µm filter.

### FANS for enrichment of dopaminergic nuclei

Flow-sorting parameters for DAPI gating are described in Martin et al. (https://www.protocols.io/view/frozen-tissue-nuclei-extraction-for-10xv3-snseq-bi62khge). For NR4A2-positive selection on a flow sorter, a DAPI versus 647 gating was established by selection of the 2.5–4.0% most highly fluorescent *NR4A2* nuclei. Flow cytometry data were analyzed, processed and visualized with Sony SH800S software.

### Generation of single-nucleus suspensions from *M. musculus*

Nuclei were isolated from mouse brain samples using a previously published^63^ protocol (https://www.protocols.io/view/frozen-tissue-nuclei-extraction-for-10xv3-snseq-bi62khge; see protocols.io link for all buffers and solution concentrations). All steps were performed on ice or cold blocks and all tubes, tips and plates were precooled for >20 min before starting isolation. Briefly, 60-µm sections of midbrain (∼50 mg) were placed in a single well of a 12-well plate and 2 ml of ExB was added to each well. Mechanical dissociation was performed by trituration using a P1000 pipette, pipetting 1 ml of solution slowly up and down with a 1-ml Rainin tip (no. 30389212), without creation of froth or bubbles, a total of 20 times. Tissue was allowed to rest in the buffer for 2 min and trituration was repeated. In total, four or five rounds of trituration and rest were performed. The entire volume of the well was then passed twice through a 26-gauge needle into the same well. Approximately 2 ml of tissue solution was transferred into a 50-ml Falcon tube and filled with WB for a total of 30 ml of tissue solution, which was then split across two 50-ml Falcon tubes (∼15 ml of solution in each tube). The tubes were then spun in a swinging-bucket centrifuge for 10 min at 600*g* and 4 °C. Following spinning, the majority of supernatant was discarded (∼500 μl remaining with the pellet). Tissue solutions from two Falcon tubes were then pooled into a single tube of ∼1,000 μl of concentrated nuclear tissue solution. DAPI was then added to the solution at the manufacturer’s (Thermo Fisher Scientific, no. 62248) recommended concentration (1:1,000).

Following sorting, nuclei concentration was counted using a hemocytometer before loading into a 10X Genomics 3’ V3 Chip.

### Generation of single-nuclei suspensions from *M. fascicularis*, *Tupaia belangeri* and *Rattus norvegicus*

Fresh frozen *R. norvegicus* and *T. belangeri* brains were mounted in a cryostat. *T. belangeri* and *R. norvegicus* nuclear isolation was adapted from a previously published protocol (https://www.protocols.io/view/frozen-tissue-nuclei-extraction-for-10xv3-snseq-bi62khge). Following nuclear isolation, *NR4A2* staining was performed on isolated nuclei at 1:350 concentration (no. sc-376984, Santa Cruz). The staining and flow-sorting parameters followed were similar to human midbrain and macaque nuclei sorting, as above. Both *NR4A2-* and DAPI-labeled nuclei were flow sorted into one sample. Following nuclei isolation, nuclei were loaded into a 10X Genomics 3’ V3 Chip.

### Generation of Slide-seqV2 libraries from *M. fascicularis*

To generate Slide-seqV2 data, *M. fascicularis* flash-frozen brain tissue samples were equilibrated in a cryostat at −20 °C and sectioned to the region of interest into 10-μm sections. Samples were transferred to the Slide-seq array puck by placing that on top of the tissue section with immediate transfer to an Eppendorf tube with hybridization buffer (6× sodium chloride sodium citrate (SSC) + 1:20 RNase Inhibitor, Lucigen). Libraries were generated according to the published Slide-seqV2 protocol: (https://www.protocols.io/view/library-generation-using-slide-seqv2-bxijpkcn). Reagents and solutions, along with oligonucleotide sequences, are listed under the Materials section in protocols.io. All steps were followed except for step 10: following removal of beads from two washes with TE-TW (10mM Tris-HCl + 1mM EDTA, Ethylenediaminetetraacetic acid, + 0.05% Tween-20), the bead pellet was immediately resuspended in 200 μl of 0.1 N NaOH.

Libraries were sequenced based on the standard Illumina protocol. Samples were pooled at a concentration of 4 nM and read structure was specified according to protocols.io. All samples were sequenced on either NovaSeq 6000 SP or S2 flowcells.

### snRNA-seq and library preparation

For all single-nuclei experiments, the 10X Genomics (v.3) kit was used according to the manufacturer’s protocol recommendations. Library preparation was performed according to the manufacturer’s recommendation. Libraries were pooled and sequenced on either a NextSeq or NovaSeq4000.

### Hybridization chain reaction on tissue sections

Postmortem human and mouse midbrain tissues flash-frozen at −80 °C were cryosectioned at −15 to −20 °C to create 12-µm sections on SuperFrost Plus slides, which were frozen at −80 °C until staining. Slides were allowed to warm up to room temperature (RT) before being placed in 4% paraformaldehyde for 15 min at RT. Slides were then washed three times with 70% ethanol for 5 min before incubation in 70% ethanol for 2 h. After incubation, slides were then incubated at 37 °C in Probe Hybridization buffer (Molecular Instruments) for 10 min in a humidified chamber. The probe solution was then freshly prepared by the addition of 0.4 pmol of each probe set (Molecular Instruments) per 100 µl of Probe Hybridization buffer. Slides were then incubated overnight at 37 °C in a humidified chamber. After 18–24 h, sections were sequentially washed in the following solutions: (1) 75% probe wash buffer and 25% 5× SSCT (SSC + 10% Tween-20), (2) 50% probe wash buffer and 50% 5× SSCT, (3) 25% probe wash buffer and 75% 5× SSCT and (4) 100% 5× SSCT. Slides were then washed for 5 min at RT in 5× SSCT and allowed to preamplify in Probe Amplification buffer (Molecular Instruments) for ≥30 min at RT. Hairpins were then freshly prepared by the addition of 1 µl of hairpin per 100 µl of Amplification Buffer (Molecular Instruments), and snap-frozen using a thermocycler with the following settings: 95° for 90 s then cooling to RT (20 °C) at a rate of 3 °C min^−1^. Snap-frozen hairpins were added to the desired volume of amplification buffer.

Following overnight incubation at RT in a humidified chamber, slides are washed twice for 30 min at RT with 5× SSCT. An appropriate amount of Fluoromount Gold plus NucBlue was added, and slides were then coverslipped and sealed with clear nail polish and stored at 4 °C until imaging. Probe names and their associated accession numbers are listed as follows: *TH* (NM_000360.4), *CALB1* (NM_001366795), *TMEM200A* (NM_001258276.1), *SOX6* (NM_001145811.2), *AGTR1* (NM_000685), *GEM* (NM_005261.4), *TRHR* (NM_003301.7), *GFRA2* (NM_001165038.2), *PART1* (NR_024617.1), *Th* (NM_009377), *Calb1* (NM_009788.4), *Fam83b* (NM_001045518.2), *Gem* (NM_010276.4) and *Acta2* (NM_007392).

### Nissl staining and imaging of macaque midbrain sections

Frozen macaque midbrain sections (10 µm) were equilibrated to RT and excess condensate wiped off. Sections were fixed in 70% ethanol for 2 min, followed by rehydration in ultrapure water for 30 s. Excess water was wiped off and slides were stained with Arcturus Histogene Solution (ThermoFisher, no. 12241-05) for 4 min. Excess dye was tapped off and slides were rehydrated in water for 10 s for destaining. Slides were sequentially fixed in 70, 90 and 100% ethanol for 30 s, 10 s and 1 min, respectively, post-fixed in xylene solution for 1 min then mounted with Fisher Chemical Permount (no. SP15-100) and coverslipped. Images were acquired with a Keyence BZ-800XE microscope and a Nikon Apo ×10 objective.

### Imaging and analysis of single-molecule FISH experiments testing for differential vulnerability of dopaminergic neurons

Imaging was performed with a Nikon Eclipse Ti microscope and a Yokogawa CSU-W1 confocal scanner unit with an Andor Zyla 4.2 Plus camera. Images were acquired using a Nikon Apo ×40/1.15 numerical aperture (NA) WI objective.

For in situ validation of DA subtype vulnerability, slides were viewed in their entirety by scanning tissue with a Nikon Apo ×40/1.15 NA WI objective. An area was considered to contain a single-positive neuron if the following criteria were met: (1) neuron at least 70% in frame; (2) DAPI signal indicating a nucleus within the major area of the neuron (defined by expression of *TH*); (3) signal in the *TH* channel that does not overlap with other channels; and (4) distinct puncta are visible. An area was considered to contain a double-positive neuron if the above criteria were met and the neuron contained between three and five distinct, nonoverlapping puncta in the 561-nm channel. An area was considered to contain a triple-positive neuron if all the above criteria were met and the neuron contained between three and five distinct, nonoverlapping puncta in the 647-nm channel.

A total of ten control and ten PD midbrains were imaged and quantified for the following sets of markers: *TH*/*AGTR1*/*SOX6* and *TH*/*CALB1*/*TMEM200A*. All manual quantification of subpopulations was performed blinded to case versus control status for both probe sets. To generate the *P* values shown in Fig. 3e, a Wilcoxon rank-sum test was performed on the fractional abundance of double- and triple-positive cells.

### Imaging and processing for stereotactic localization of dopaminergic neuron subtypes

Imaging was performed with a Nikon Eclipse Ti microscope and a Yokogawa CSU-W1 confocal scanner unit with an Andor Zyla 4.2 Plus camera. Images were acquired using a Nikon Apo ×40/1.15 NA WI objective. Images were acquired at 80% laser power and 300-ms exposure. The number of fields to be imaged in the *x* and *y* axes was determined by manual testing of various amounts until the entire visible substantia nigra was contained within the area to be imaged.

Images were converted into composite images and, using the criteria defined above for in situ validation of proportional alterations, were sorted depending on whether they contained neurons using FIJI. A binary mask was created using the Nikon NIS Elements Advanced Research software and applied to images based on thresholding intensity, area and circularity. The 3D Objects Counter function in FIJI was applied to the binary masks for each image, and detected masks were then multiplied by the other two channels and cells manually annotated. A minimum volume value of 5,000 from the 3D Objects Counter analysis was used to remove autofluorescent puncta and sections of DA neurons that were partially cut. For the low-resolution view of a single midbrain slice (Fig. 2f), we performed a scan at 488 nm with a Nikon Apo ×10 objective using a BZ-X Series Keyence microscope on a single adjacent section to the slides, to quantify and localize subtypes. The circled white region roughly corresponds to the curved area of high DA neuron density found in the localization mask images (Fig. 2g).

### Preprocessing of snRNA-seq reads

Sequencing reads from human midbrain experiments were demultiplexed and aligned to the hg19 reference using DropSeqTools v.2.4.0 (https://github.com/broadinstitute/Drop-seq) with default settings. Reads from species were aligned with the following genomes: rat (Rnor_6.0.fasta), mouse (mm10), macaque (M_fascicularis_5.0.fasta) and tree shrew (treeshrew_2.0.fasta). Digital count matrices were subsequently generated from DropSeqTools. Sequencing reads from human caudate nucleus experiments were demultiplexed and aligned to the hg19 reference using CellRanger v.3, with default settings, and counts were generated using the ‘count’ function. Seqeuncing reads from the macaque snRNA-seq experiment were generated using CellRanger v.5.

### Preprocessing of Slide-seqV2 sequencing reads

The *M. fascicularis* Slide-seqV2 pucks were demultiplexed, aligned to the M_fascicularis_5.0.fasta reference and matched to the spatially encoded bead barcodes using Slide-seq tools pipeline v.0.2 (https://github.com/MacoskoLab/slideseq-tools) and PuckCaller package (https://github.com/MacoskoLab/PuckCaller/), with default settings.

### Identification of candidate midbrain dopaminergic neuron TFs for enrichment strategy

To nominate potential nuclear TFs for flow-based enrichment, we used a recently published comprehensive survey of the mouse brain^22^. We downloaded midbrain data and performed differential expression between DA neurons and all other cell types using a Wilcoxon rank-sum test from the presto package (https://github.com/immunogenomics/presto). We intersected our list with a list of mouse TFs and determined AUC values.

### Cell type clustering and annotation of human datasets

Cell types were defined using a two-step process. First, individuals were clustered independently to extract major cell types using a modified version of the Seurat v.2 (ref. ^64^) workflow. Briefly, for each individual we determined a UMI cutoff based on manual inspection and identification of an inflection point on a knee plot, the rank-ordered total number of UMIs per droplet. Across all datasets we used a minimum of 650 UMIs per cell as our cutoff. For each individual, the gene-by-cell digital gene expression (DGE) matrix was column normalized, values multiplied by 10,000 and subsequently log-normalized. We used the Seurat v.2 procedure to find a list of highly variable genes with default cutoff and subsetted the DGE on this highly variable gene list. The resulting subsetted DGE was scaled and principal component analysis (PCA; *k* = 30) was performed. Finally, *t*-distributed stochastic neighbor embedding was performed on the determined low-dimensional, 30-component embedding, and clusters were identified by Louvain clustering on the defined low-dimensional, 30-component embedding. On the first round, we removed those cells with a high fraction of mitochondrial reads (>10% of reads mapping to mitochondrial genes)^65^, those that had substantially lower numbers of UMIs relative to the rest of the dataset and putative doublets (clusters that showed substantial expression of marker genes from two or more major cell types). Clusters were annotated to one of seven major cell types (astrocytes, non-dopaminergic neurons, dopaminergic neurons, oligodendrocytes, oligodendrocyte precursor cells (OPCs), endothelial cell/pericytes and microglia/macrophages) based on per-cluster expression of a list of marker genes identified in a published mouse midbrain scRNA-seq dataset^22^. Once major cell types were extracted, subtypes were defined by joint integrative analysis across individuals as described below.

We used Harmony^66^ to integrate non-neuronal cell types across individuals into a shared space by removal of batch effects while preserving biological variation. The following analysis steps were performed for each non-neuronal cell type, separately. First, we searched for highly variable genes in each of 18 individual-level datasets using a variance-stabilizing transformation method from the Seurat package in R. Genes identified as highly variable in at least four individual-level datasets were selected for PCA analysis. Next, individual-level datasets were combined after standardization of the expression of highly variable genes to have a mean of zero and variance of one. We performed PCA analysis with PCs weighted by the variance they explained using Seurat. To integrate datasets, a grid search was performed on the number of PCs and harmony parameter sets to find a solution with optimal mixing of cells from different subjects while maximizing the separation of different cell states as judged by the expression patterns of marker genes. The integrated space was used to construct the nearest-neighbor graph. Clustering was performed using the smart local moving (SLM) algorithm with a resolution of 0.8.

For neuronal cell types, the LIGER projection method was used to minimize the influence of the diseased cell transcriptome on the reduced space. Briefly, neuronal nuclei from eight neurologically normal individuals were clustered and annotated using the standard LIGER workflow. Cells were normalized by dividing by the total number of UMIs and subsequently scaled. Variable genes were selected followed by integrative, non-negative matrix factorization and quantile normalization. Next, profiles from individuals with PD/LBD were integrated by projecting nuclei onto the integrative non-negative matrix factorization (iNMF) dimensions generated from control individuals by setting ‘projection = T’ in the iNMF algorithm step. Clusters were defined by the SLM algorithm at a resolution of 0.6, and marker genes were generated using a Wilcoxon rank-sum test comparing nuclei from one cluster against the remainder of the nuclei. At the subtype level we examined those cells showing expression of more than one cell type, removing these putative doublets. Alignment score calculations in LIGER were performed using the default package settings. We additionally aligned DA subtypes using Harmony (Extended Data Fig. 2h), utilizing the same strategy employed for our non-neuronal analyses and finding consistent annotations as our LIGER analysis (Extended Data Fig. 2i,j). For *P* values plotted on density distributions in Extended Data Fig. 2i, we performed a hypergeometric test on the nearest-neighbors assignment generated from a *k*-nearest-neighbors graph determined from the Harmony low-dimension space for each cluster (where a successful draw in this case is a nearest neighbor being assigned to the same cluster as the cell being tested).

In clustering our DA subtypes, we noticed that SOX6_DDT had higher expression of genes such as *UBB*, as well as for oxidative phosphorylation and heat shock protein components (Extended Data Fig. 2b). These RNAs are heavily translated, and are hence enriched in rough endoplasmic reticulum (ER) that may be differentially retained during nuclear dissociation^67^. Nonetheless, SOX6_DDT also showed enrichment of genes not related to potential dissociation artifacts. Indeed, the marker *DDT* itself encodes for an enzyme involved in melanin production^68^, suggesting that some aspects of this population may reflect a biologically distinct type. We hypothesize that these cells may have more melanin, or a different chemical composition of melanin deposits, that render cytoplasmic ER components more prone to remaining stuck to dissociated nuclei. The heritability enrichment in SOX6_AGTR1 was robust to the removal of the SOX6_DDT cluster (Bonferroni-corrected MAGMA *P* = 0.018 without SOX6_DDT versus Bonferroni-corrected MAGMA *P* = 0.027 with SOX6_DDT), and did not change the significance of our differential abundance analysis using MASC, nor for the SOX6_AGTR1 population (OR = −0.82, FDR-adjusted *P* < 0.05) or CALB1_GEM or CALB1_TRHR (OR = 1.46 and 0.84, respectively, FDR-adjusted *P* < 0.05 for both clusters).

### SCENIC analysis to identify differentially regulated regulons

To identify differentially regulated regulons associated with specific dopaminergic subtypes, we employed SCENIC with user-recommended settings from the SCENIC vignette (http://htmlpreview.github.io/?https://github.com/aertslab/SCENIC/blob/master/inst/doc/SCENIC_Running.html). Briefly, we averaged the log-normalized gene values of single-nucleus profiles from our dataset of non-PD control individuals per dopaminergic subtype. We filtered on genes that had at least one count per bulk profile across all ten subtypes. We ran a correlation analysis using GENIE3 and then ran SCENIC to determine TF modules within these correlations. Using AUCell, we scored cells based on regulon activity and plotted these scaled regulon scores on a per-dopaminergic subtype basis. To determine statistically significant differences in regulon activity, we ran a Wilcoxon rank-sum test (using the presto package https://github.com/immunogenomics/presto) on regulon scores between major dopaminergic subtypes and ranked cells based on their AUC values and adjusted *P* < 0.05. This same workflow was performed on annotated macaque DA subtypes (determined from integrative analysis comparing macaque and human profiles; Extended Data Fig. 5d).

### Robust cell type decomposition analysis of Slide-seqV2 data

To assign cell types from the single-nucleus reference of *M. fascicularis* to Slide-seqV2 pucks, we used the robust cell type decomposition (RCTD^33^) package. RCTD allows for spatial pixels to be assigned sparse mixtures of the reference cell types, which is congruous to Slide-seqV2’s near-single-cell resolution.

The macaque reference annotations used are detailed below (‘integration of dopaminergic neurons across species’). We combined high-level annotation with the more detailed subtype definition of DA subtypes, based on an integrative analysis with our reference human control dataset (Extended Data Fig. 5d; see below). The gene set used in RCTD was created by combining a reference-wide set of highly variable genes (first 5,000 genes under a variance-stabilizing transformation) with those selected in neuronal cell type identification. Further, Slide-seqV2 pucks were filtered to retain only those beads with at least 50 UMIs and 20 genes.

To aid RCTD in identification of beads with artifactual signatures, we added a ‘cell type’ to the reference that corresponded to cells that did not pass standard quality control metrics and that expressed high levels of *UBB*, and oxidative phosphorylation genes heavily enriched in rough ER, which may be differentially retained in nuclei dissociation preparations^67^. Beads assigned by RCTD solely to this artifact classification were discarded from later analysis.

The mapping was performed with RCTD’s doublet-aware mode, which is designed to most accurately decompose cells captured by Slide-seqV2’s ten micron beads. RCTD classifies each bead as a mixture of reference cell types; additionally, it categorizes these classifications as ‘singlet’, ‘doublet uncertain’, ‘doublet certain’ or ‘reject’, depending on the number of cell types assigned and assignment confidence. We filtered out ‘reject’ beads and used classifications from the other three categories for later analysis.

To delineate each puck’s medial–lateral midline (for plotting the distances of subtypes CALB1 and SOX6), *TH* and *CALB1* gene expression were plotted and points manually chosen on the boundary between each gene’s expression to calculate a line of best fit.

### Integration of dopaminergic neurons across species

To jointly define dopaminergic neurons across species, we used LIGER’s projection method. Briefly, we ran a modified version of the Seurat v.2 (ref. ^64^) workflow to identify major cell types across all nonhuman species. Gene-by-cell DGE matrices were normalized and centered. Variable gene selection was performed, followed by non-negative matrix factorization (NMF), Louvain clustering and uniform manifold approximation and projection (UMAP) projection based on low-dimensional embedding from NMF. Low-quality nuclei were defined as those with >10% of their reads mapping to mitochondrial transcripts, and putative doublets were defined by coexpression of one or more major cell type markers identifiable from a recently published study of mouse midbrain^22^. DA neurons from all species were subsetted based on expression of clusters with high levels of *TH* and/or *SLC18A2* (for tree shrew, which did not have the gene *TH* annotated as such in the reference genome). To produce the multispecies integrative analysis shown in Fig. 1g, we first integrated mouse and human DA neurons using LIGER with the following parameters: (1) number of variable genes, 1,080; (2) *k* = 10 (number of latent factors); (3) lambda = 5 (strength of integration); (4) knn_k (number of nearest neighbors) = 45; and (5) SLM resolution (number of clusters) = 1.5. We then projected on rat, tree shrew and macaque DA neurons using the online iNMF branch of LIGER, with the setting ‘projection = TRUE’. After quantile normalization, datasets were jointly clustered using the Louvain clustering algorithm in LIGER with a resolution of 1.5. Joint low-dimension embedding was visualized using the UMAP algorithm.

To define a reference snRNA-seq dataset for our RCTD analysis of Slide-seq data from macaque midbrain, we projected DA neurons from *M. fascicularis* onto our neurotypical human control dataset (Extended Data Fig. 5d). To accomplish this, we subsetted our macaque midbrain data to DA neurons only (defined by expression of *TH*, *SLC6A3* and *SLC18A2*). Macaque DA neuron profiles were then projected onto the human neurotypical control reference map using the online iNMF branch of LIGER, with the setting ‘projection = TRUE’. After quantile normalization, datasets were jointly clustered via a nearest-neighborhood graph (knn_k = 45, number of nearest neighbors), and clusters were determined using the Louvain clustering algorithm in LIGER with a resolution of 1.2. Joint low-dimension embedding was visualized using the UMAP algorithm. One cluster with high levels of mitochondrial and oxidative phosphorylation genes was flagged for removal, but was used for RCTD mapping analysis (see above). Putative doublets (defined by the coexpression of one or more major cell type markers identifiable from a recently published study of mouse midbrain^22^) were also removed from integrative analysis. DA subtypes were assigned to macaque nuclei based on the nearest-neighbor graph and coclustering with human DA cell types.

### Differential abundance assessment of cell types in association with PD and other covariates

To identify differentially abundant cell subpopulations in association with PD, we employed MASC^69^. MASC is a generalized, mixed-effect model with a binomial distribution that determines whether a certain covariate significantly influences the membership of a given nucleus. We included the following fixed covariates in the generalized, mixed-effect model: sex, status (control or disease) and *NR4A2* (whether nuclei were captured in a NR4A2-positive or -negative library). Finally, the library from which the nuclei were sampled was included as a random effect to account for the intralibrary correlation of cell numbers. Cell subpopulations were considered significant at FDR-adjusted *P* < 0.05 and absolute odds ratio >0.

### Disease enrichment embedding score

To project information about relative abundance onto the reduced dimension space for DA subtypes, we developed an enrichment score (Fig. 3c). Briefly, we binned the two-dimensional embedding for both disease and control individuals and, for each bin, we determined the total number of cells per individual within that bin. We then averaged those values across individuals per bin for both case and control, then scaled these scores across all bins to provide a normalized estimate of the relative abundance of certain DA subtypes across the UMAP space.

### Differential expression analysis

To identify differentially expressed genes across all major cell types, we employed model-based analysis of single-cell transcriptomes (MAST)^70^. MAST is a mixed-effect hurdle model that models droplet-based, single-nucleus/cell expression data as a mixture of a binomial and normal distribution (for log-normalized nonzero expression values) while systematically accounting for predefined covariates. We included the following fixed-effect covariates in our model: log(number of UMIs), sex, percentage of reads that map to mitochondrial genes per nucleus (percent.mito) and status (control or disease) to test the effect of the disease on each cell type. We additionally included the library from which a nucleus was sampled as a random-effect covariate in the model, to account for intralibrary correlation of expression data. To evaluate the effect of disease on expression, we performed differential expression analysis across all major cell types. For all non-neuronal cell types we used only cells from NR4A2-negative libraries; for neurons, we used only *NR4A2-*positive libraries. Genes were defined as significantly differentially expressed at FDR < 0.05 using Benjamini–Hochberg correction. For all expression tests we used the discrete coefficient of MAST to determine the coefficient estimate of the effect of disease on expression.

To identify differentially expressed genes between clusters, we evenly downsampled the control datasets across eight individuals to 100,000 nuclei and performed MAST comparisons between cell types and subtypes while accounting for batch variation. We included all the same covariates as above (except for disease status), and included additional covariates of cell type identity and a covariate for whether the cell derived from a *NR4A2*-positive or -negative library. To create marker gene sets, we selected genes with coefficient >0 and FDR-adjusted *P* < 0.05.

### Heritability enrichment analyses

To determine which cell types and subtypes are enriched for heritable risk of traits, we used MAGMA^40^ (https://ctg.cncr.nl/software/magma) and stratified LD score regression (s-LDSC)^43^. For AD and PD, we downloaded publicly available summary statistics from the most recently available studies^39,42^. We then performed a single-nucleotide polymorphism to GENE calculation with either the MAGMA tool or the equivalent webserver (FUMA: https://fuma.ctglab.nl/) using default parameter settings. For all major cell types/subtypes, we took the top 3,500 marker genes ranked by *z*-score as determined from MAST (see above) for each cell type and subtype as a defined gene set. For 17 subtypes we were unable to reach sufficient nuclei to acquire 3,500 marker genes and, instead, used the maximum number of genes identified that reached statistical significance (Supplementary Table 10). Importantly, despite setting upper bounds on the maximum number of differentially expressed genes for these smaller cell types, there was no correlation with the number of nuclei sampled and *P* values from MAGMA for our DA subtypes (Extended Data Fig. 10c). We ran MAGMA on the set annotation setting to test the significance of whether the marker gene set of that cell type was enriched for heritable risk of the trait. The resulting *P* values were Bonferroni corrected for multiple hypothesis testing across major cell types and subtypes.

To run a stratified LD score for partitioning of heritability across cell type gene sets, we followed the standard procedure defined by the s-LDSC wiki (https://github.com/bulik/ldsc/wiki/Cell-type-specific-analyses). Briefly, we first munged all summary statistics with the munge_sumstats.py script provided with the s-LDSC package. We next defined a reference gene set based on all genes expressed in our substantia nigra dataset. We specified baseline weights based on the model defined for cell-type-specific analyses^43^. Finally, we ran partitioned heritability on the gene sets across all cell types and subtypes. Resulting *P* values were Bonferroni corrected for multiple hypothesis testing across major cell types and subtypes.

To create the ‘pseudo-Manhattan’ plots in Fig. 4e, we ordered the nominated MAGMA genes by their *z*-score and the −log(*P* values) from our MAST analysis. GO signatures (Fig. 4f) for top genes that drive the association of the SOX6^+^ signature with the common variants of PD were determined by taking all genes higher than MAST *z* > 4.568. We used enrichR^48^ on these genes to determine enriched GO biological processes using the GO_Biological_Process_2017 library.

### Familial PD variant enrichment analysis

To test whether there exists any enrichment of genes previously nominated as containing variants that cause familial PD, we gathered a list of known familial PD genes from a recent whole-exome sequencing study^38^. We ran Fisher’s exact test using the geneOverlap package between genes that were considered specifically expressed in a major cell type, as determined by the top 10% of genes ordered by the AUC metric with the Wilcoxon rank-sum test, and these 26 genes. The resulting *P* values were then corrected for multiple hypothesis testing using the Bonferroni method (eight tests for major cell types).

### GSEA of TF gene sets across dopamine subtypes

To identify TFs and their downstream targets as enriched, we used GSEA from the fGSEA package^46^. The choice of fGSEA on the list of differentially expressed genes over a SCENIC-based approach was motivated by recent studies suggesting the use of random-effect models (as we have carried out via MAST) to control for false discovery in single-cell expression data with multiple replicates. We first gathered all TFs from three TF regulon libraries in the enrichR^48^ database: TRRUST, ENCODE and ARCHS4. We then ran GSEA with the following parameters—minsize = 1, maxsize = 500, nperm = 1,000—to test the enrichment of a TF targets list in an ordered list of differentially expressed genes per subtype. All *P* values were FDR corrected for hypothesis testing across the ten subtypes tested.

### Regression analyses

To determine the lines of best fit for Extended Data Fig. 10c (number of nuclei sampled versus heritability enrichment significance), we performed an ordinary least-squares regression on −log_10_-transformed *P* values against the number of nuclei sampled per cluster for *P* values generated by both MAGMA and s-LDSC. A Wald test was performed to assess whether the absolute value of the coefficient of the slope of the line was significantly >0.

To determine any bias associated with case-control status on the median number of UMIs per library, we used a linear mixed-effect model and included covariates of case-control status as a fixed effect and the library as a random effect (Extended Data Fig. 7h). To determine the influence of year of collection on the quality of sampling, we regressed the median number of UMIs acquired per library against the year of collection for each tissue sample (Extended Data Fig. 7i).

### Statistics and reproducibility

No statistical methods were used to predetermine sample sizes, but our sample sizes are similar to those reported in previous publications using single-cell analyses to identify vulnerable and resistant cell populations (PMID: 33432193). No randomization occurred during the study. Given the differences in age and sex across the two arms of the study, those covariates were included in all differential abundance and expression calculations. For in situ validation of selective neuronal susceptibility and resistance and localization/quantification of DA subtypes, all samples were blinded before staining, imaging and quantification. Blinding was not performed for any other experiment as the single-nucleus RNA-seq data were generated agnostic to the hypothesis identified in the study. For age/PMI (post-mortem interval) comparisons and log(*P* value) comparisons with nuclei sampled, data were considered normally distributed but this was not formally tested. For other statistical tests, justification of the distribution was either unneeded (in the case of nonparametric methods) or previously provided in the referencing literature^69,70^.

### Reporting Summary

Further information on research design is available in the Nature Research Reporting Summary linked to this article.

## Online content

Any methods, additional references, Nature Research reporting summaries, source data, extended data, supplementary information, acknowledgements, peer review information; details of author contributions and competing interests; and statements of data and code availability are available at 10.1038/s41593-022-01061-1.

## Supplementary information

Supplementary Information Supplementary Figs. 1–3.

Reporting Summary

Supplementary Tables Table 1: Basic demographics for eight neuropathologically normal midbrains. Table 2: Basic demographics for ten PD/LBD individuals whose midbrains were sampled. *Note: midbrain loss scores (if reported by brain bank) range 0–3 (0, none; 3, severe). Table 3: Demographics and neuropathology for ten control and ten PD frozen midbrains used for in situ validation of selective vulnerability. Table 4: Basic demographics for four neuropathologically normal individuals for human caudate samples. Table 5: In situ quantification of raw values for CALB1/TMEM200A experiment (left) and SOX6/AGTR1 experiment (right). Table 6**:** Number of dopaminergic neurons sampled per individual. First column indicates donor ID used in this study, second denotes the number of dopaminergic neurons sampled and the third indicates the status of donors (control versus PD). Table 7: Aggregate summary statistics for cell subtypes stratified by case/control status. First column indicates status and the second indicates the cell subtype, where text preceding the underscore delimiter indicates the major cell type of which the subtype is a part. Median_number_of_UMIs, median number of UMIs. Cluster_size, number of nuclei sampled per subtype. Table 8: Results from MAST differential expression analysis for ten dopaminergic subtypes. DA_subtype, dopaminergic neuron subtype; primerid, gene name; *Pr*(>Chisq), *P* values from MAST-H (linear mixed-effect model, two-sided test); Coef, MAST-D coefficient; Ci.hi/ci.lo, upper and lower bounds of confidence interval for coefficient estimate; *Z*, *z*-score from MAST; fdr, Benjamini-Hochberg-adjusted *P* value. Table 9: Raw *P* value results from MAGMA and s-LDSC heritability enrichment analyses. First column indicates the method used. Celltype/subtype indicates the results of major cell type and subtype enrichment for each trait. dSPN, direct spiny projection neurons; iSPN, indirect spiny projection neurons; P.value, *P* value obtained from MAGMA regression or s-LDSC test (two-sided). Table 10: Summary statistics of number of differentially expressed genes obtained for each cell subtype. Number of marker genes identified by MAST analysis for each cell subtype at the thresholds specified in the title of the second column.

## Data Availability

All processed data, UMAP coordinates and annotations have been made freely available to download and inspect at the Broad Institute Single Cell Portal (note, two links, one for single-nucleus data and the other for Slide-seq data): https://singlecell.broadinstitute.org/single_cell/study/SCP1768/ and https://singlecell.broadinstitute.org/single_cell/study/SCP1769/. Raw and processed data to support the findings of this study have been deposited in GEO under accession no. GSE178265. For TF analysis, the TRRUST 2019, Encode and CHEA Consensus and ARCHS4 TF-coexpression public datasets were used. All are available for download via the enrichR website: https://maayanlab.cloud/Enrichr/#libraries

## Code availability

All code used to generate the main figures of this paper can be found at https://github.com/tkamath1/Kamathetal2022
