# Supplementary material for: Single-cell genomic profiling of human dopamine neurons identifies a population that selectively degenerates in Parkinson’s disease
Source: Nat Neurosci. 2022 May 5;25(5):588–95. doi: 10.1038/s41593-022-01061-1 (PMC9076534; doi:10.1038/s41593-022-01061-1)
Supplement: Supplementary file 1 — Supplementary Figs. 1–3. [file 41593_2022_1061_MOESM1_ESM.pdf]

nature portfolio

https://doi.org/10.1038/s41593-022-01061-1

Supplementary information

Single-cell genomic profiling of human dopamine neurons identifies a population that selectively degenerates in Parkinson's disease

In the format provided by the authors and unedited

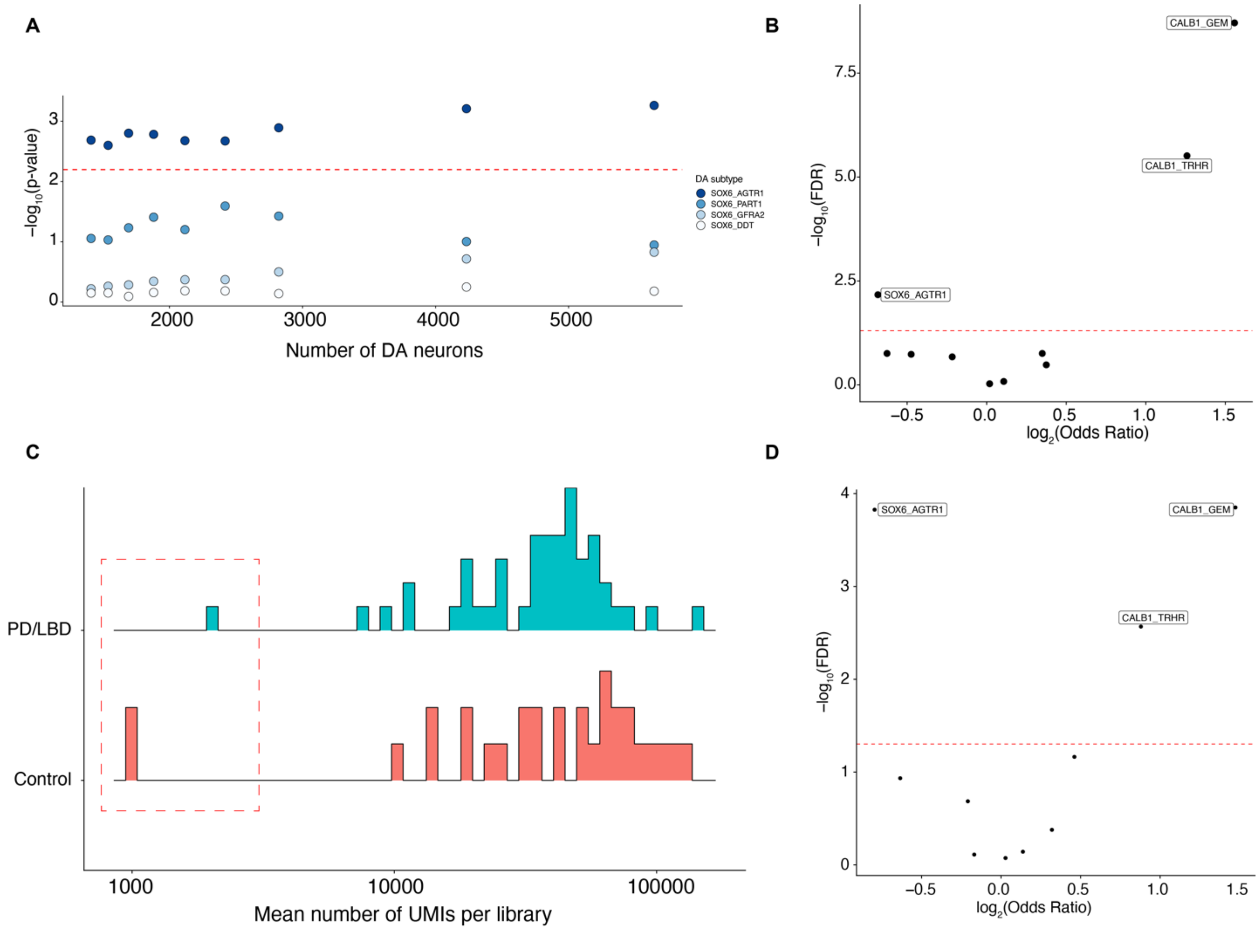

Supplementary Fig. 1: Robustness of DA proportional analyses A, Dot plot of downsampling analysis for *SOX6*+ DA subtypes. X-axis denotes size of dataset after downsampling and Y-axis is the MASC-computed -$\log_{10}$ p-values associated with disease status. Red dotted line is FDR-adjusted p-value = 0.05. Dots are colored by subtype. **B,** Volcano plot of MASC results for DA subtypes using only the PD cases and neurotypical controls. Red dotted line represents FDR significance ($p < 0.05$). Dots are labeled if they reach FDR significance < 0.05. **C,** Log plot of mean number of UMIs per library stratified by PD/LBD and control. Dotted red line identifies 3 libraries with a lower mean number of UMIs. **D,** Volcano plot of MASC results for DA subtypes with three low-quality libraries identified in (**C**) removed. Red dotted line represents FDR significance ($p < 0.05$). Dots are labeled if they reach FDR significance < 0.05.

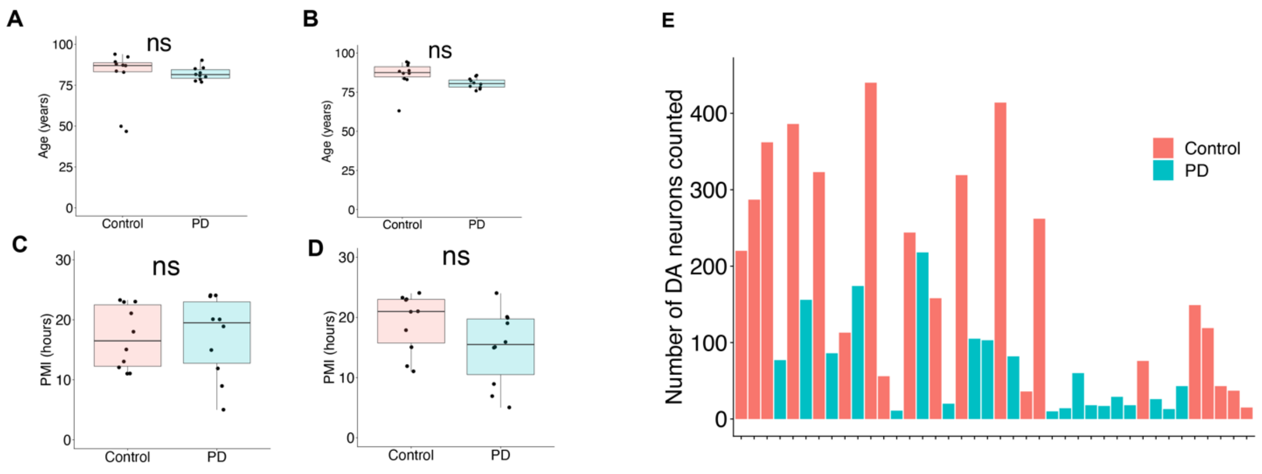

Supplementary Fig. 2: Imaging analysis of highly vulnerable and resistant DA subtypes in the SNpc by smFISH. A,B,C,D, Box plots, stratified by disease status, of age of death (**A,B**) and postmortem interval (**C,D**), for each subject in the *in situ* quantification of *TH+/CALB1+/TMEM200A+* cells (**A,C**) and *TH+/SOX6+/AGTR1+* cells (**B,D**) (n = 10 neurotypical controls and n = 10 PD postmortem samples for all boxplots **A**-**D**). For box plots in (**A-D**), center bars indicate median value and lower and upper hinges correspond to first and third quartiles respectively. Whisker distance from upper and lower hinges represent no more than 1.5*interquartile range (ns = not significant, Welch's two-sample t-test, p = 0.72 for (**A**), p = 0.10 for (**B**), p = 0.95 for (**C**), p = 0.12 for (**D**)). **E,** Total number of DA neurons counted per slide; bars are colored by disease status.

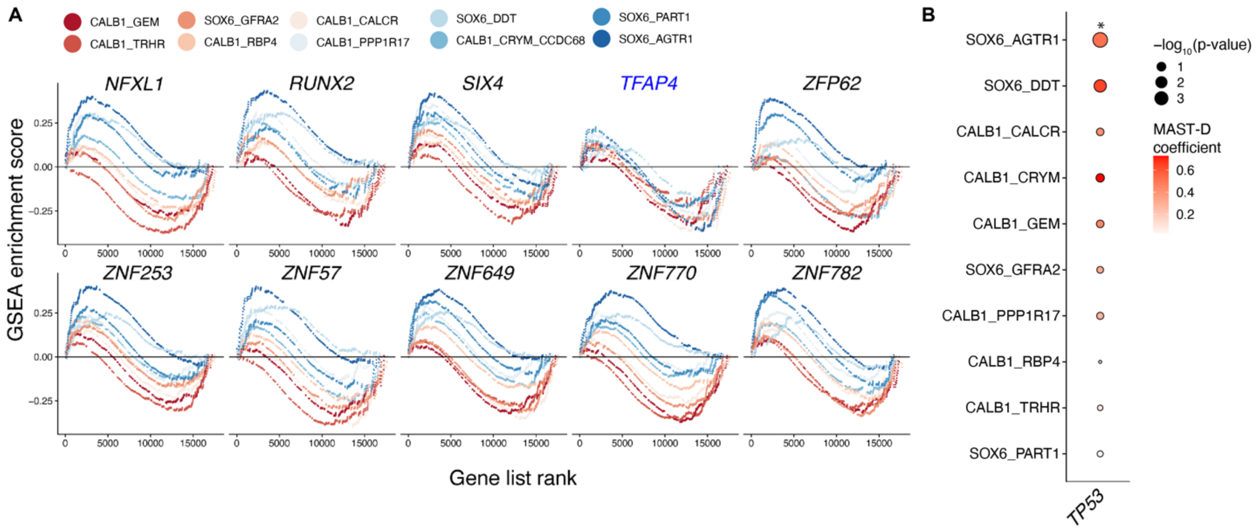

Supplementary Fig. 3: Additional gene set enrichment analysis (GSEA) trace plots for transcription factor analysis. **A,** GSEA trace plots for additional transcription factors that reached significance within the SOX6_AGTR1 population but no other DA subtypes (Methods). Colors correspond to odds ratios derived from MASC analysis (Fig. 3B, Methods). **B,** Dot plot of *TP53* differential expression results for 10 DA subtypes. Size of dots corresponds to -$\log_{10}$-transformed p-values and color corresponds to MAST-D coefficient (see Methods). * = indicates significantly differentially expressed (FDR-adjusted p-value < 0.01).
