# Supplementary material for: Single-cell genomic profiling of human dopamine neurons identifies a population that selectively degenerates in Parkinson’s disease
Source: Nat Neurosci. 2022 May 5;25(5):588–95. doi: 10.1038/s41593-022-01061-1 (PMC9076534; doi:10.1038/s41593-022-01061-1)
Supplement: Supplementary file 2 — Reporting Summary [file 41593_2022_1061_MOESM2_ESM.pdf]

nature portfolio

Corresponding author(s): Evan Macosko

Last updated by author(s): 03/16/2022

Reporting Summary

Nature Portfolio wishes to improve the reproducibility of the work that we publish. This form provides structure for consistency and transparency in reporting. For further information on Nature Portfolio policies, see our Editorial Policies and the Editorial Policy Checklist.

Statistics

For all statistical analyses, confirm that the following items are present in the figure legend, table legend, main text, or Methods section.

n/a	Confirmed	
☐	☒	The exact sample size (*n*) for each experimental group/condition, given as a discrete number and unit of measurement
☐	☒	A statement on whether measurements were taken from distinct samples or whether the same sample was measured repeatedly
☐	☒	The statistical test(s) used AND whether they are one- or two-sided<br>*Only common tests should be described solely by name; describe more complex techniques in the Methods section.*
☐	☒	A description of all covariates tested
☐	☒	A description of any assumptions or corrections, such as tests of normality and adjustment for multiple comparisons
☐	☒	A full description of the statistical parameters including central tendency (e.g. means) or other basic estimates (e.g. regression coefficient) AND variation (e.g. standard deviation) or associated estimates of uncertainty (e.g. confidence intervals)
☐	☒	For null hypothesis testing, the test statistic (e.g. *F*, *t*, *r*) with confidence intervals, effect sizes, degrees of freedom and *P* value noted<br>*Give P values as exact values whenever suitable.*
☒	☐	For Bayesian analysis, information on the choice of priors and Markov chain Monte Carlo settings
☒	☐	For hierarchical and complex designs, identification of the appropriate level for tests and full reporting of outcomes
☐	☒	Estimates of effect sizes (e.g. Cohen's *d*, Pearson's *r*), indicating how they were calculated

Our web collection on statistics for biologists contains articles on many of the points above.

Software and code

Policy information about availability of computer code

Data collection	Human, mouse, tree shrew, and rat raw single-nuclei sequencing data were processed, aligned, and converted to a digital gene expression matrix by DropSeqTools v2.4.0 with default settings. Macaque single-nuclei raw sequencing data were processed, aligned, and converted to a digital gene expression matrix by CellRanger v5. The human caudate nuclei experiment sequencing data were processed, aligned, and converted to a digital gene expression matrix with CellRanger v3. Slide-seq raw sequencing data were processed, aligned, and converted to a digital gene expression and x-y coordinate matrices using SlideSeqTools v0.2. In situ hybridization data was acquired with Nikon NIS Elements AR software.
Data analysis	All data was analyzed using Seurat v2.3.4, LIGER v0.4.2, SCENIC v1.2.4, RCTDv1.0, MASTv1.8.2, MASC v0.1.0-alpha, MAGMAv1.8, LDSCv1.0.1, Harmony v1.0, fGSEAv3.14, and SLMv1.3.0 on R v3.6.3. In addition, all scripts and custom code to reproduce the main figures are available at the following github repo: https://github.com/tkamath1/Kamathetal2022. All in situ hybridization images were analyzed with ImageJ v2.1.0/1.53c and Nikon NIS Elements AR software. The Sony SH800S software was used to analyze, visualize, and process flow cytometry data.

For manuscripts utilizing custom algorithms or software that are central to the research but not yet described in published literature, software must be made available to editors and reviewers. We strongly encourage code deposition in a community repository (e.g. GitHub). See the Nature Portfolio guidelines for submitting code & software for further information.

Data

Policy information about availability of data

All manuscripts must include a data availability statement. This statement should provide the following information, where applicable:

- Accession codes, unique identifiers, or web links for publicly available datasets
- A description of any restrictions on data availability
- For clinical datasets or third party data, please ensure that the statement adheres to our policy

All processed data, UMAP coordinates, and annotations have been made freely available to download and visualize at the Broad Institute Single Cell Portal (note two links, one for the single-nuclei data and the other for the Slide-seq data): https://singlecell.broadinstitute.org/single_cell/study/SCP1768/ and https://singlecell.broadinstitute.org/single_cell/study/SCP1769/. Raw and processed data to support the findings of this study have been deposited in GEO under accession number GSE178265. For transcription factor analysis, the TRRUST 2019, Encode and CHEA Consensus, and ARCHS4 TF-coexpression public datasets were used. All are available for download via the enrichR website: https://maayanlab.cloud/Enrichr/#libraries

Field-specific reporting

Please select the one below that is the best fit for your research. If you are not sure, read the appropriate sections before making your selection.

- [x] Life sciences
- [ ] Behavioural & social sciences
- [ ] Ecological, evolutionary & environmental sciences

For a reference copy of the document with all sections, see nature.com/documents/nr-reporting-summary-flat.pdf

Life sciences study design

All studies must disclose on these points even when the disclosure is negative.

Sample size	No statistical methods were used to pre-determine sample sizes but our sample sizes are similar to those reported in previous publications using single-cell analyses to identify vulnerable and resistant cell populations (PMID: 33432193)
Data exclusions	For transcriptomic data, a UMI cutoff of 650 was set to remove low-quality nuclei from the analysis.
Replication	For differential abundance assessment, we performed in situ hybridization to validate the selective loss of the SOX6+/AGTR1+ DA neurons. The identification of a loss of this cell population was nominated by our differential abundance analysis and validated by an orthogonal assay using single-molecule FISH repeated once across an external set of 10 PD and 10 neurotypical control postmortem samples and performed once. Experiments to generate single-nuclei RNA-sequencing enriched DA neurons were validated across postmortem human and macaque tissue, having been repeated 19 times, 18 times using postmortem human samples, and a single time with a macaque postmortem sample by a wholly different experimentalist.
Randomization	No randomization occurred during the study. Given differences in age and sex across two arms of the study, those covariates were included in all differential abundance and expression calculations.
Blinding	For in situ validation of selective neuronal susceptibility and resistance and the localization/quantification of DA subtypes, all samples were blinded before staining, imaging, and quantification. Blinding was not performed for any other experiment as the single-nuc. RNA-sequencing data were generated agnostic to the hypothesis identified in the study.

Reporting for specific materials, systems and methods

We require information from authors about some types of materials, experimental systems and methods used in many studies. Here, indicate whether each material, system or method listed is relevant to your study. If you are not sure if a list item applies to your research, read the appropriate section before selecting a response.

Materials & experimental systems

n/a	Involved in the study	
[ ]	[x]	Antibodies
[x]	[ ]	Eukaryotic cell lines
[x]	[ ]	Palaeontology and archaeology
[ ]	[x]	Animals and other organisms
[ ]	[x]	Human research participants
[x]	[ ]	Clinical data
[x]	[ ]	Dual use research of concern

Methods

n/a	Involved in the study	
[x]	[ ]	ChIP-seq
[ ]	[x]	Flow cytometry
[x]	[ ]	MRI-based neuroimaging

Antibodies

Antibodies used	1 - anti-NR4A2-A647 antibody (Santa Cruz, sc-376984, A647)<br>2 - anti-NeuN-PE; clone A60 (EMD Millipore Corp., FCMAB317PE)
Validation	anti-NR4A2-647 validation from Santa Cruz website (https://www.scbt.com/p/nurr1-antibody-f-5)<br>"[anti-NR4A2] available conjugated to...Alexa Fluor® 647 for WB (RGB), IF, IHC(P) and FCM"<br>Anti-Neun-PE validation from EMD website (https://www.emdmillipore.com/US/en/product/Milli-Mark-Anti-NeuN-PE-Antibody-clone-A60,MM_NF-FCMAB317PE?ReferrerURL=https%3A%2F%2Fwww.google.com%2F#overview)<br>"This Milli-Mark Anti-NeuN-PE Antibody, clone A60 is validated for use in FC (flow cytometry) for the detection of NeuN."

Animals and other organisms

Policy information about studies involving animals; ARRIVE guidelines recommended for reporting animal research

Laboratory animals	Animals used:<br>Mus musculus - C57BL/6J, 3 months, male/female<br>Rattus norvegicus - 0.2 years, male<br>Tupaia belangeri - 3 years, male<br>Macaca fascicularis - 8 years, male<br>Mice were group housed with a 12-hour light-dark schedule and allowed to acclimate to their housing environment for two weeks post arrival. For mus musculus housing, ambient temperature was strictly maintained between 68 and 72 degrees Fahrenheit and humidity was strictly maintained between 30 and 50%. All procedures involving animals at MIT were conducted in accordance with the US National Institutes of Health Guide for the Care and Use of Laboratory Animals approved by the Massachusetts Institute of Technology Committee on Animal Care. All procedures involving animals at the Broad Institute were conducted in accordance with the US National Institutes of Health Guide for the Care and Use of Laboratory Animals under protocol number 0120-09-16.
Wild animals	No wild animals were used in this study.
Field-collected samples	No field-collected samples were used in this study.
Ethics oversight	All housing and procedures involving rodents were conducted in accordance with the US National Institutes of Health Guide for the Care and Use of Laboratory Animals under protocol number 0129-09-16 and approved by the Broad Institute Committee on Animal Care (IACUC) All non-human primate (NHP) tissue was processed in compliance with the Broad Institute IBC (IBC#: 2016-00127). All human tissue falls under a "Not Engage" designation determined by the Broad Institute IACUC (Federal-wide assurance #: FWA00014055).

Note that full information on the approval of the study protocol must also be provided in the manuscript.

Human research participants

Policy information about studies involving human research participants

Population characteristics	This study involved the generation of RNA-sequencing data from postmortem human midbrain samples which fall under a "Not Engage" policy (NE5200) approved by the Broad IACUC (Federal Wide Assurance number: FWA00014055). Human midbrain samples age and biological sex are listed in Extended Data Tables 1-4. No genotypic information was obtained on these samples. Past diagnoses are listed in Extended Data Tables 1 and 2.
Recruitment	No recruitment was performed for this study.
Ethics oversight	This study falls under a "Not Engaged" determination as approved by the Broad Institute IACUC (FWA00014055).

Note that full information on the approval of the study protocol must also be provided in the manuscript.

Flow Cytometry

Plots

Confirm that:

☒ The axis labels state the marker and fluorochrome used (e.g. CD4-FITC).

☒ The axis scales are clearly visible. Include numbers along axes only for bottom left plot of group (a 'group' is an analysis of identical markers).

☒ All plots are contour plots with outliers or pseudocolor plots.

☒ A numerical value for number of cells or percentage (with statistics) is provided.

Methodology

Sample preparation	All samples were prepared according to the Methods section of this paper. A detailed protocol for single-nuclei isolation is available via Protocols.io at: https://www.protocols.io/view/frozen-tissue-nuclei-extraction-for-10xv3-snseq-bi62khge. Nuclei

isolates were obtained from cryosections of postmortem mammalian tissue.

Instrument	Samples were flow-sorted using a Sony SH800 Cell Sorter
Software	The standard Sony SH800S software was used for flow cytometry analysis
Cell population abundance	Abundances of the NR4A2+ neurons were determined post-sorting using single-nucleus RNA-sequencing
Gating strategy	No preliminary FSC/SSC gating was performed. All gating is performed as listed in the Methods of the paper and the associated protocols.io link for the DAPI gating: https://www.protocols.io/view/frozen-tissue-nuclei-extraction-for-10xv3-snseq-bi62khge?step=19.

☒ Tick this box to confirm that a figure exemplifying the gating strategy is provided in the Supplementary Information.
